# Leveraging Diamines to Unlock the Mn‐MACHO Catalyst in the Reduction of CO_2_ to Methanol

**DOI:** 10.1002/anie.202524012

**Published:** 2026-01-27

**Authors:** Mohamed E. A. Safy, Raquel J. Rama, Niklas F. Both, David Balcells, Kathrin Junge, Matthias Beller, Ainara Nova

**Affiliations:** ^1^ Hylleraas Centre for Quantum Molecular Sciences Department of Chemistry University of Oslo Oslo 0315 Norway; ^2^ Center for Materials Science and Nanotechnology (SMN) Department of Chemistry University of Oslo Oslo 0315 Norway; ^3^ Leibniz‐Institut für Katalyse e.V. Albert‐Einstein‐Straße 29a 18059 Rostock Germany

**Keywords:** Amidation reaction, CO_2_ hydrogenation, Metal‐ligand cooperative catalysis, Microkinetic modelling, Reaction mechanism

## Abstract

The conversion of CO_2_ into energy‐dense liquid fuels, such as methanol, represents a cornerstone of sustainable chemistry; however, most homogeneous catalytic systems still rely on noble metals or Lewis acid additives. Here, we report the first protocol for amine‐assisted CO_2_ hydrogenation to methanol using a Mn–MACHO catalyst without any Lewis acid co‐catalyst, achieving turnover numbers up to 45.2, the highest reported for Mn systems. A combined computational, microkinetic, and experimental study reveals that diamines dramatically enhance activity compared to monoamines by promoting a highly exergonic double amidation step. This thermodynamic driving force shifts the equilibrium away from formate resting states toward the active catalyst, thereby accelerating methanol formation. The correlation established between amidation free energies (Δ*G*_amidation_) and methanol productivity provides a rational design principle for tailoring amine promoters across Ru‐ and Mn‐based MACHO catalysts. These insights advance the development of sustainable, base‐metal‐catalyzed CO_2_ conversion strategies and open opportunities for integrated carbon capture and utilization.

## Graphical abstract

CO_2_ hydrogenation to methanol using a Mn–MACHO catalyst was achieved without Lewis acid promoters, delivering TONs up to 45.2. Diamines, by driving a highly exergonic double amidation reaction, divert formate resting states toward the active catalyst. The correlation between Δ*G*_amidation_ and methanol productivity enables the rational design of the amine for novel Ru and Mn‐MACHO catalysis.

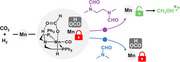

CO_2_ hydrogenation to energy‐dense liquid products, particularly methanol, is pivotal for achieving a carbon‐neutral methanol economy.^[^ ^1^, ^2^, ^3^, ^4^, ^5^, ^6^ ^]^ Homogeneous catalysts, especially the PNP‐pincer Ru‐MACHO‐Ph (**Ru‐1‐Ph**, Scheme 1), have demonstrated effective CO_2_ hydrogenation to methanol under mild conditions.^[^ ^6^, ^7^, ^8^, ^9^, ^10^, ^11^, ^12^ ^]^ The utilization of amines as a co‐catalyst for this process plays a crucial dual role: enhancing the CO_2_ capture and facilitating the hydrogenation process to produce methanol.^[^ ^13^, ^14^ ^]^ Notably, polyamines are the most efficient, while monoamines exhibit minimal or no CO_2_ conversion to methanol.^[^ ^15^, ^16^ ^]^ In this work, this preference has been rationalized and used to achieve the direct CO_2_ hydrogenation to methanol with Mn complexes without the need of Lewis acid additives to remove the formate from the catalyst.^[^ ^17^, ^18^, ^19^ ^]^ The use of a base metal catalyst provides a sustainable and cost‐effective pathway toward scalable CO_2_‐to‐methanol technologies.

**Scheme 1:**
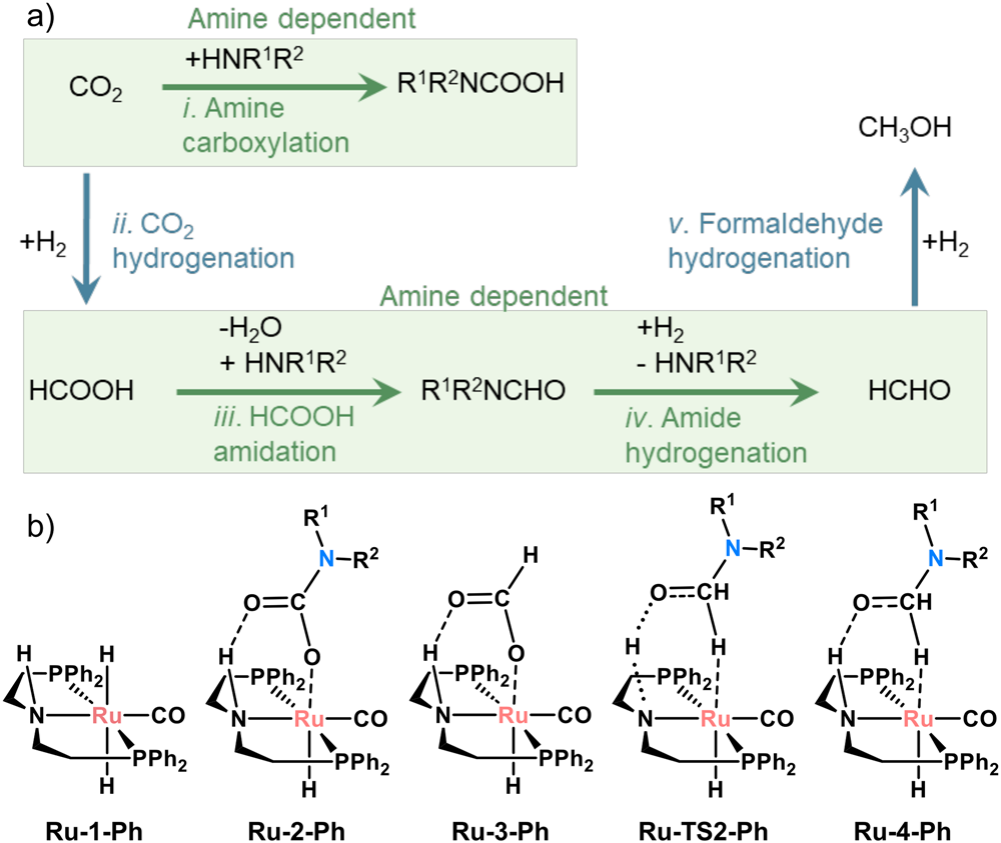
a) Overview of reactions and b) relevant intermediates in the amine‐assisted CO_2_ hydrogenation by **Ru‐1‐Ph**. R_1_ = CH_3_ and R_2_ = CH_2_CH_2_NCH_3_CHO.

Scheme 1 summarizes the primary reaction steps involved in the amine‐assisted CO_2_ hydrogenation to methanol, catalyzed by the Ru‐MACHO‐Ph complex (**Ru‐1‐Ph**, Scheme 1). CO_2_ can either react with amine, forming carbamate (reaction i) or with hydrogen, yielding formic acid (reaction ii).^[^ ^20^ ^]^ The reaction of this product with amine yields formamide, which is then hydrogenated to formaldehyde, regenerating the amine (reactions iii and iv). Finally, formaldehyde undergoes hydrogenation to yield methanol (reaction v). From these steps, i, iii, and iv involve amines and could, therefore, be responsible for the higher activity observed with polyamines compared to monoamines.

To understand the enhanced activity observed with polyamines, we performed a computational study at the DFT and DLPNO‐CC levels of theory, using the SMD method to model the solvent effects of tetrahydrofuran.^[^ ^17^, ^18^ ^]^ Dimethylethylenediamine (**D1**, Figure 1) was chosen as a representative polyamine, balancing high catalytic activity with computational feasibility. The results obtained were then compared to those of the monoamine dimethylamine (**M1**, Figure 1) reported in our previous study.^[^ ^16^ ^]^

**Figure 1:**
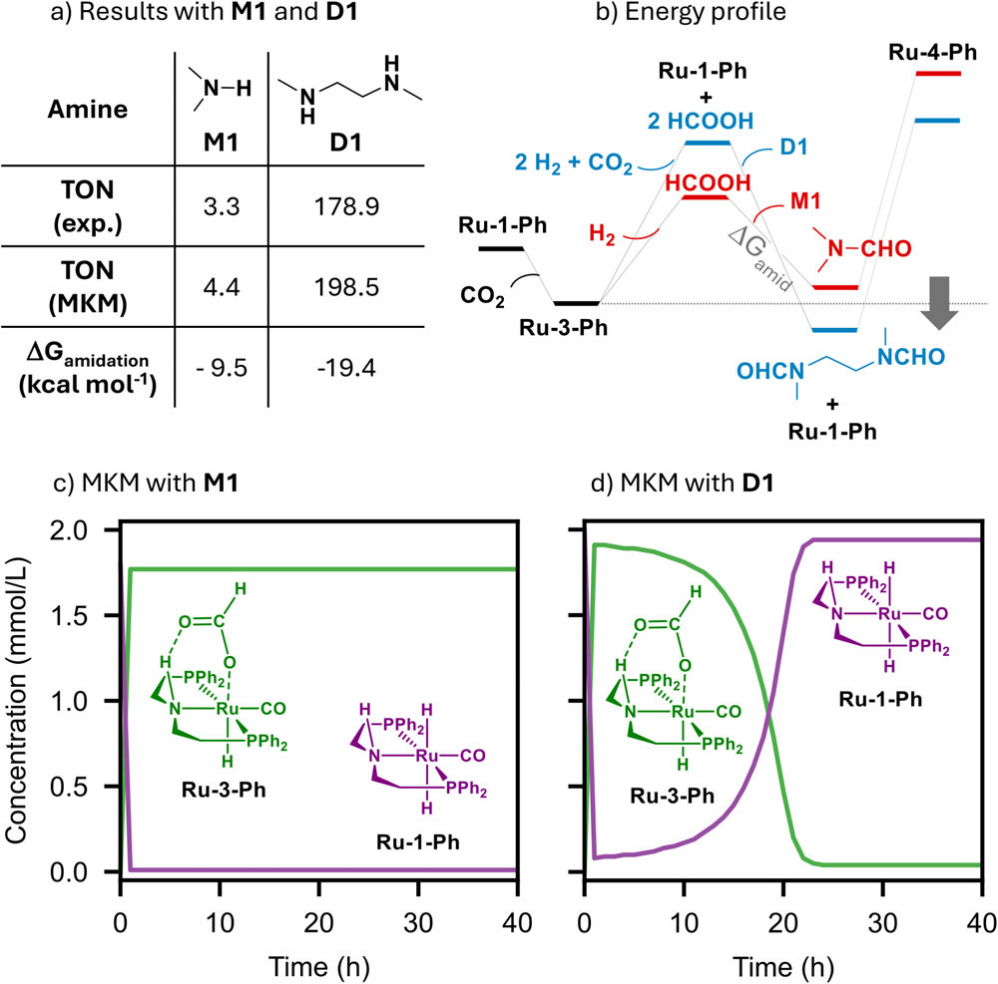
a) Table containing the TON of methanol obtained experimentally and with the MKM, and the Δ*G*_amidation_ for **M1** and **D1**. b) Schematic energy profile explaining the easier catalyst recovery and higher activity with **D1** (see Figures S11 and S12 for Ru and Mn profiles with Gibbs free energies). c) and d) concentration of **Ru‐1‐Ph** and **Ru‐3‐Ph** (in mmol L^−1^) over time (in hours) using the MKM with **M1** and **D1**, respectively.

The first step we investigated was the formation of Ru‐carbamate (**Ru‐2‐Ph**, Scheme 1), as it has been proposed to be one of the catalyst resting states of the reaction, together with Ru‐formate (**Ru‐3‐Ph**).^[^ ^16^, ^20^ ^]^ One of the differences between mono‐ and di‐amines is the ability of diamines to react with two CO_2_ molecules, leading to mono‐ and di‐carbamates. Therefore, the formation of Ru‐carbamate with each of these species was calculated using **Ru‐1‐Ph**, CO_2_, **M1,** and **D1** as energy references. The DLPNO‐CC results (Figure S2) show minor energy differences (0.2 kcal mol^−1^) for the carbamate with **M1** (**Ru‐2‐Ph‐M1)** and dicarbamate with **D1** (**Ru‐2‐Ph‐D1‐d)**, which become higher (ca. 3 kcal mol^−1^) for the monocarbamate with **D1** (**Ru‐2‐Ph‐D1‐m)**. Since a lower energy for the catalyst resting state indicates a slower reaction, this step could not account for the higher methanol yield observed with diamines.

Next, we focused on the hydrogenation of formamide, which has been proposed to be the rate‐limiting step in the CO_2_ to methanol reaction.^[^ ^16^, ^21^ ^]^ The DFT free energy profile for the hydrogenation of the formamides derived from **D1** and **M1** to their corresponding hemiaminal is shown in Figure S3, with selected DLPNO‐CC energies in Scheme S1. Similarly, to the carbamate results, minor energy differences of up to 1.5 kcal mol^−1^ were found in the transition state, **Ru‐TS2‐Ph**, and **Ru‐4‐Ph** intermediate (Scheme 1b). In addition, the energies of the formamide derived from **D1** were again the highest. Hence, the energies obtained for the hydrogenation of formamide with **M1** and **D1** also failed to explain the disparate activities of mono‐ versus di‐amines.

To gain a better understanding of the reaction, a microkinetic model (MKM) for the **D1**‐assisted CO_2_ hydrogenation to methanol was constructed using DLPNO‐CC energies and compared to experimental kinetics, as well as to our model for **M1**.^[^ ^16^ ^]^ Figure 1a summarizes the experimental turnover number (TON), MKM‐predicted TON, and the amidation free energy (Δ*G*_amidation_, reaction iii in Scheme 1a).

The simulated MKM yielded methanol TONs (4.4 for **M1** and 198.5 for **D2**) very close to the experimental (3.3 for **M1** and 178.9 for **D2**) for both amines. Also, the MKM predicted a negligible amount of formamide intermediate for **D1** (Figures S4–S7), which is consistent with the experimental results in the literature.^[^ ^15^ ^]^ The only significant difference in the MKMs is the Δ*G*_amidation_ (−9.5 and −19.5 kcal mol^−1^ for **M1** and **D2**, respectively). This reaction has a similar Gibbs free energy for **M1** and **D1** when only one formic acid equivalent is added (Δ*G* = −9.5 kcal mol^−1^ for **M1** and 9.9 kcal mol^−1^ for **D1**). However, it is preferred by 9.5 kcal mol^−1^ if **D1** reacts with two molecules of formic acid, yielding a di‐amide. This energetic preference favors the formation of **Ru‐1‐Ph** and formamide over the formate complex **Ru‐3‐Ph**, despite the most endergonic formation of two formic acid molecules from CO_2_ and H_2_ (Figure 1b). This scheme is consistent with the fastest consumption of the formate complex in the MKM using **D1**, when compared to **M1** (Figure 1c,d). It also suggests that the larger the Δ*G*_amidation_ is, the more displaced this equilibrium is. However, at a certain amidation energy this step becomes less relevant, due to the equilibrium becoming entirely displaced. This result was also supported by decreasing the Δ*G*_amidation_ in the MKM of **D1** from −1 to −15 kcal mol^−1^, which shows a lack of methanol increase after lowering the Δ*G*_amidation_ of **D1** by 3 kcal mol^−1^ (Figure S10). Notably, doubling the **M1** initial concentration in the MKM increased the TON of methanol from 4.4 to only 4.9, confirming that the significantly larger TON with **D1** is not arising from higher amine concentration.

To further support this hypothesis, we calculated the Δ*G*_amidation_ at the DFT level for 15 amines, including eight monoamines (**M2**‐**M9**) and seven diamines (**D1‐D7**), previously tested experimentally by Prakash et al. for the CO_2_ hydrogenation to methanol with **Ru‐1‐Ph** (Figures 2a, S8, and S9) under the same reaction conditions.^[^ ^15^ ^]^ The correlation between experimental methanol production (in mmol) and the calculated Δ*G*_amidation_ is shown in Figure 2b.

**Figure 2:**
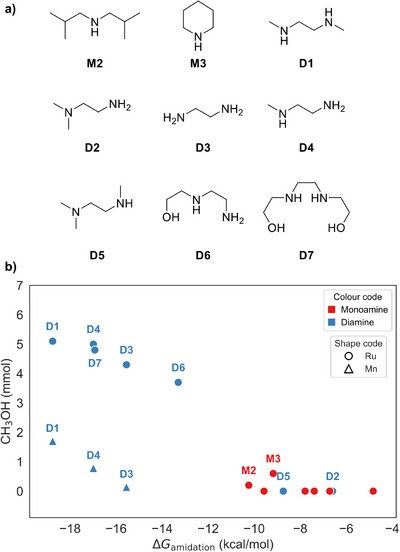
a) Molecular structures of selected investigated amines. b) Correlation between the DFT Gibbs free energy of formamide formation (Δ*G*_amidation_) and the amount of methanol produced (mmol) for the investigated amines (see Figure S13 for linear fitting). Experimental conditions: CO_2_/H_2_ = 1:3 (75 bar) at 145 °C for 20 h in triglyme (10 ml), with the total amine functionality held constant at 30.6 mmol^[^ ^15^ ^]^ for **Ru‐BH‐Ph** (10 µmol) (Figure 3a), and CO_2_/H_2_ = 1:7 (80 bar) at 145 °C for 40 h in dioxane (20 mL) with 4 mmol of amine **Mn‐Br‐Ph** (40 µmol).

The points in Figure 2b can be separated in two groups depending on the number of nitrogen atoms, shown in different colors (red for monoamines, and blue for diamines). The only exceptions are diamines **D2** and **D5,** which contain a tertiary amine that cannot react with formic acid yielding formamide and therefore behave like monoamines. There is thus a clear correlation between methanol formation and Δ*G*_amidation_. The more negative the Δ*G*_amidation_, the higher the methanol formation. Among monoamines, only **M2** and **M3** produced methanol (TON = 0.2 and 0.6, respectively): **M2** formed the most stable formamide (Δ*G*_amidation_ = −10.2 kcal mol^−1^), whereas **M3**, despite a less stable formamide with Δ*G*_amidation_ = −9.2 kcal mol^−1^, has the lowest formamide hydrogenation energy barrier attributable to its cyclic structure.^[^ ^20^ ^]^

This result indicates that amidation energies larger than −10.0 kcal mol^−1^ will not yield methanol at the investigated temperature under the applied conditions, unless a cyclic amine facilitates the hydrogenation of formamide, as it occurs with **M3**. In the case of primary and secondary diamines, we found a nearly linear correlation between Δ*G*_amidation_ and methanol formation, indicating the important influence of the amidation step in these cases.

Despite the high catalytic activity achieved by Ru‐based catalysts, their sustainability and cost‐effective scalability remain challenging. Aiming to replace Ru with a base metal, we experimentally tested whether the use of **D1** could enable CO_2_ hydrogenation to methanol with **Mn‐Br‐Ph**, without the need for a Lewis acid to react with formate.^[^ ^17^, ^18^ ^]^ To our delight, the formation of methanol was achieved with TONs of up to 45.2 after 40 h (see Supporting Information). A decrease in the TON was observed when moving from **D1** to **D4** and **D3** (from 45.2 to 19.5 and 3.4, respectively), as expected due to their less negative Δ*G*_amidation_. These TONs correspond to 1.81, 0.78, and 0.14 mmol of methanol, showing a more pronounced slope (−0.49 compared to −0.28) in the Δ*G*_amidation_ versus methanol correlation than with Ru (Figures 2b and S13, including linear fitting).

Next, we tested the catalytic activity using **D1** and Mn complexes containing *i*Pr and Cy substituents in the phosphine (**Mn‐Br‐iPr** and **Mn‐Br‐Cy**), as they have been shown to influence the methanol formation with Ru.^[^ ^15^ ^]^ Interestingly, as in the case of Ru, **Mn‐Br‐Ph** showed the highest activity with a TON = 45.2, while **Mn‐Br‐Cy** showed the lowest (TON = 14.0) (Figure 3). For comparison, the same reaction conditions were used to test the reactivity with **Ru‐BH‐Ph** and **Ru‐Cl‐Cy**, yielding TONs of 178.9 and 48.5, respectively.

**Figure 3:**
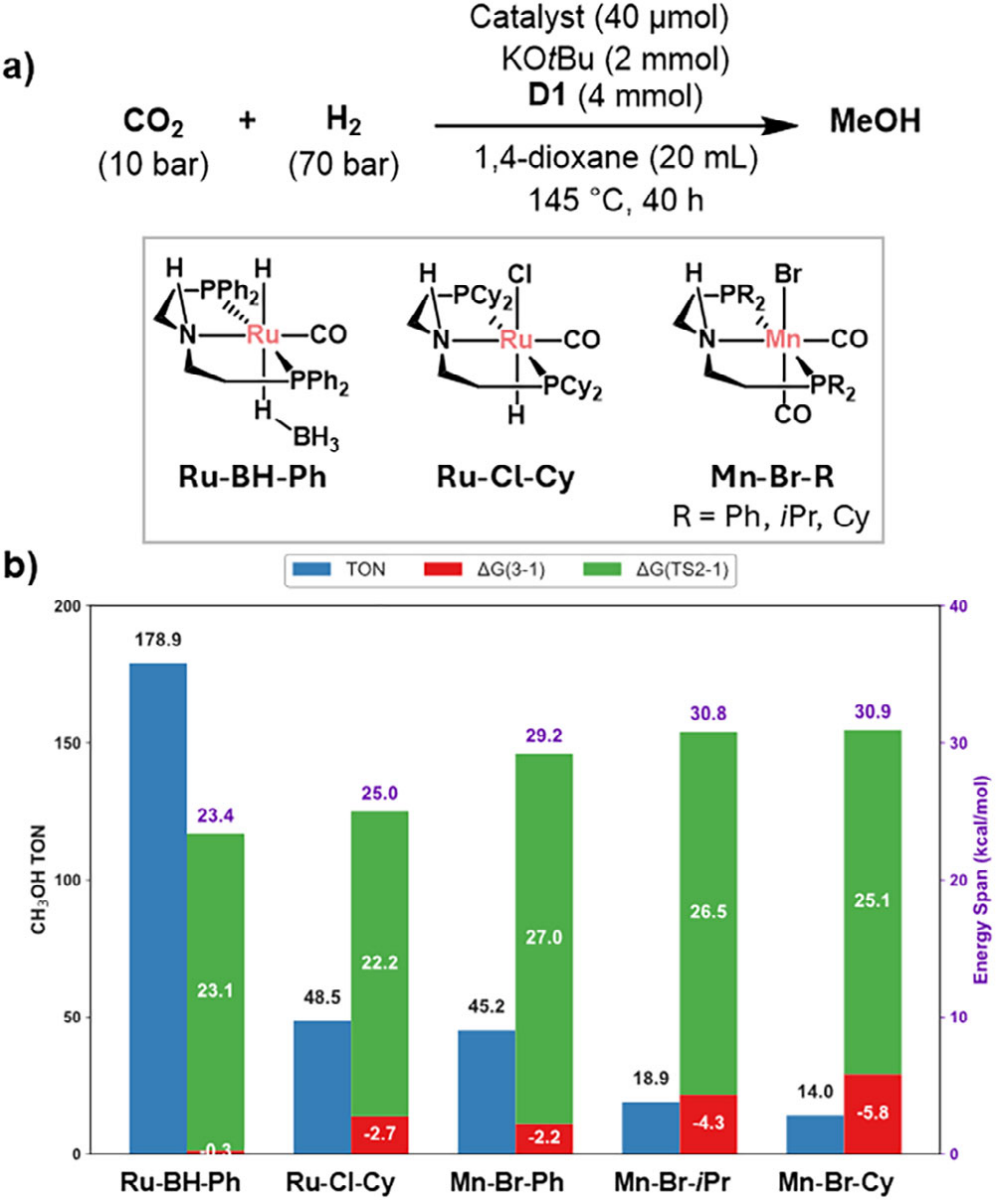
a) Experimental conditions for CO_2_ hydrogenation to methanol. **D1** stands for dimethylethylenediamine. b) Comparison of experimental methanol turnover numbers (TON, blue bars, left axis) using the optimized reaction conditions and the pre‐catalyst in the Supporting Information and energy spans calculated in DFT (kcal mol^−1^, red and green bars, right axis) for CO_2_ hydrogenation to methanol with different catalysts.

In a previous study using a microkinetic model, we found that small variations in the energy of **Ru‐TS2‐Ph** and **Ru‐3‐Ph** have the highest impact on the methanol formation. Therefore, we defined the energy span as the energy difference between these two species. To evaluate if the same energy span accounts for the changes in reactivity of the different substituents with Mn and Ru, we calculated the energy difference between **M‐TS2‐R** and **M‐3‐R** (M = Mn, R = *i*Pr, Cy, and Ph, and M = Ru, R = Cy, and Ph) for the experimentally tested systems. To reduce computational cost, dimethylformamide was used to model the formamide derived from **D1**, as they showed similar energy barriers with the **Ru‐1‐Ph** system. The calculated energy spans follow the order **Ru‐Ph** << **Ru‐Cy** ≈ **Mn‐Ph** < **Mn‐iPr** ≈ **Mn‐Cy**, with energies of 24.1 and 28.5 kcal mol^−1^ for the Ru systems, 29.3 kcal mol^−1^ for **Mn‐Ph**, and close to 31.0 kcal mol^−1^ for both **Mn‐iPr**, and **Mn‐Cy**. These energies are consistent with the methanol TONs being in the opposite order: the higher the energy span, the lower the methanol TON. Each energy span can be divided into two Δ*G* contributions: one corresponding to the M‐formate formation (**M‐3‐R** → **M‐1‐R** + CO_2_, Δ*G* (3–1) in red) and one corresponding to the transition state for formamide hydrogenation (**M‐TS2** → **M‐1‐R** + formamide, Δ*G* (TS2‐1) in green). This analysis shows that while the stability of the formate increases in the order Ph < *i*Pr < Cy, the opposite occurs with the energy barrier (**TS2**) in formamide hydrogenation, with no differences observed with Ru. This result explains why the most efficient systems for formamide hydrogenation (**M‐Cy**) are not the most efficient for CO_2_ hydrogenation (**M‐Ph**) for M = Ru^5^ and Mn (Figure 3).

In conclusion, we have reported for the first time a protocol for the CO_2_ hydrogenation to methanol using a Mn catalyst and dimethylethylenediamine, notably without the need for any Lewis acid co‐catalyst. The production of methanol was found to correlate with the Gibbs free energy of the amidation step, providing a clear thermodynamic rationale for the superior performance of diamines compared to monoamines. The enhanced activity originates from a double amidation reaction that shifts the equilibrium from the formate resting state toward the active catalyst and formamide intermediates. This result may be extended to other metal complexes in which M‐formate is the catalyst resting state, as with Fe‐MACHO.^[^ ^22^, ^23^ ^]^ Furthermore, the energy span between the formamide hydrogenation barrier and the formate intermediate (**M‐TS2‐R** and **M‐3‐R**) was shown to correlate with methanol productivity and to depend on the electronic properties of the phosphine substituents. Together, these results establish a mechanistic framework that links amine structure, thermodynamics, and catalyst design, thereby offering guiding principles for the rational development of base‐metal MACHO catalysts for integrated carbon capture and conversion to methanol.

## Conflict of Interests

The authors declare no conflict of interest.

## Supporting information



Supporting Information

Supporting Information

## Data Availability

The data that support the findings of this study are available in the supplementary material of this article.
