# Supplementary material for: Leveraging Diamines to Unlock the Mn‐MACHO Catalyst in the Reduction of CO2 to Methanol
Source: Angew Chem Int Ed Engl. 2026 Jan 27;65(10):e24012. doi: 10.1002/anie.202524012 (PMC12955513; doi:10.1002/anie.202524012)
Supplement: Supplementary file 1 — Supporting Information [file ANIE-65-e24012-s002.docx]

Leveraging Diamines to Unlock the Mn-MACHO Catalyst in the Reduction of CO_2_ to Methanol

Mohamed E. A. Safy,^a^ Raquel J. Rama*,^b^ Niklas F Both,^c^ David Balcells,^a^ Kathrin Junge,^c^ Matthias Beller*,^c^ Ainara Nova*^ab^

^a^Hylleraas Centre for Quantum Molecular Sciences, Department of Chemistry, University of Oslo, 0315, Oslo, Norway.

^b^Center for Materials Science and Nanotechnology (SMN), Department of Chemistry, University of Oslo, 0315 Oslo, Norway.

^c^Leibniz-Institut für Katalyse e.V. Albert-Einstein-Straße 29a, 18059 Rostock, Germany

*E-mail: a.n.flores@kjemi.uio.no; matthias.beller@catalysis.de; r.j.rama@smn.uio.no

**Table of Contents**

[**Computational methods** 2](#_Toc217027466)

[**Experimental details** 3](#_Toc217027467)

[**General considerations** 3](#_Toc217027468)

[**Optimization of reaction conditions for the CO_2_ hydrogenation to methanol** 3](#_Toc217027469)

[**Catalytic testing** 4](#_Toc217027470)

[**Mechanistic study for CO_2_ hydrogenation to methanol** 5](#_Toc217027471)

[**Carbamate complex formation** 5](#_Toc217027472)

[**Formamide hydrogenation to formaldehyde** 7](#_Toc217027473)

[**Comparison of Ru-4-Ph-D1-d and Ru-4-Ph-M1 using DLPNO-CC energies.** 9](#_Toc217027474)

[**Microkinetic modeling** 9](#_Toc217027475)

[**ΔG_amidation_ of the investigated amines** 13](#_Toc217027476)

[**Role of amine in Ru-3-Ph activation** 16](#_Toc217027477)

[**Linear fitting for the correlation between the ΔG amidation and methanol formation** 19](#_Toc217027478)

[**Cartesian coordinates of the investigated amines** 20](#_Toc217027479)

[**References** 31](#_Toc217027480)

# **Computational methods**

All the amines and their corresponding formamides were sent for a conformational search using the conformer-rotamer ensemble sampling tool (CREST, version 12) with the GFN2-xTB method [1]. All the generated conformers by CREST were refined based on electronic energies using the M06/def2-TZVP level of theory. For amines that yielded more than 50 conformers, the full conformational search and refinement protocol was repeated three times, and the lowest-energy structure was selected for further analysis. The conformers with the lowest energy were then optimized to minima using the M06L-D3 functional and the def2-SVP basis set. Frequency calculations at the same level of theory were performed to ensure that the optimized structures were minima. Single-point energy calculations were conducted using the M06-D3 method and the def2-TZVP basis set. The SMD model was employed to simulate tetrahydrofuran (THF) solvent for all the DFT calculations. All the DFT calculations were carried out using Gaussian16[2] and will be referred to as the DFT method in the next sections. ORCA 6 was used to calculate the single-point energy (in SMD-THF) of the optimized structure at the DLPNO-CCSD(T)/cc-PVTZ-dk level of theory[3], which will be referred to as the DLPNO-CC method.

To construct microkinetic models (MKM) for the Ru-MACHO-Ph system, we used CC-level energies, as recommended in our earlier work[4]. All intermediates and transition states used in the MKM were subjected to the previously reported conformational search protocol[4], in which the lowest-energy conformer among the 50 most stable conformers using DFT energies was chosen for DFT optimizations and CC calculations. Finally, the Complex Pathway Simulator (COPASI) software was used to build the microkinetic model [5].

In energy span model calculations, only the M-1-R species underwent the conformational search protocol. The most stable M-1-R structures were used to build M-3-R and M-TS2-R, which were then optimized at the M06L-D3/def2-SVP level of theory without further conformational search. The energies in the energy span model were calculated using DFT at the M06-D3/def2-TZVP method.

# **Experimental details**

## **General considerations**

All preparations and manipulations were carried out under an oxygen-free nitrogen atmosphere, using conventional Schlenk techniques. Solvents were rigorously dried and degassed before use. The manganese complexes (**Mn-Br-*i*Pr**) was prepared according to the reported procedure[6]. Reagents were purchased and used without purification. GC analyses were carried out on an Agilent 7890A chromatograph equipped with an HP-5 column (30 x 0.25 x 0.25 m) and measured with the following temperature program: 50/8-120/0/15-200/0/25-300/10 using 1,3,5-trimethoxybenzene as internal standard.

## **Optimization of reaction conditions for the CO_2_ hydrogenation to methanol**

Optimization of the amount of **D1** in the CO_2_ hydrogenation to methanol using **Mn-Br-*i*Pr** (Figure S1). In a glovebox, **Mn-Br-*i*Pr** (2 mg, 4 µmol) was weighed into a glass vial equipped with a magnetic stir bar, and 1,4-dioxane (2 mL) was added, followed by K_3_PO_4_ (42.5 mg, 0.2 mmol). The reaction mixture was stirred for approximately 5 minutes. The amine (0.2-3.4 mmol) was added, and the glass vial was closed with a screw cap with a septum and transferred into a 300 mL autoclave. The septum was punctured with a needle to allow for gas exchange, and the autoclave was flushed ten times with 5-10 bar CO_2_ before it was charged with 10 bar CO_2_ and 70 bar H_2_. The autoclave was placed in a preheated aluminum block at 145 ^o^C for 40 h. After the reaction time, the autoclave was cooled to room temperature in an ice bath and the gas pressure was carefully released. A defined amount of 1,3,5-trimethoxybenzene was added as the internal standard. The reaction mixture was diluted with THF (4 mL), filtered through Celite, and analyzed by GC.


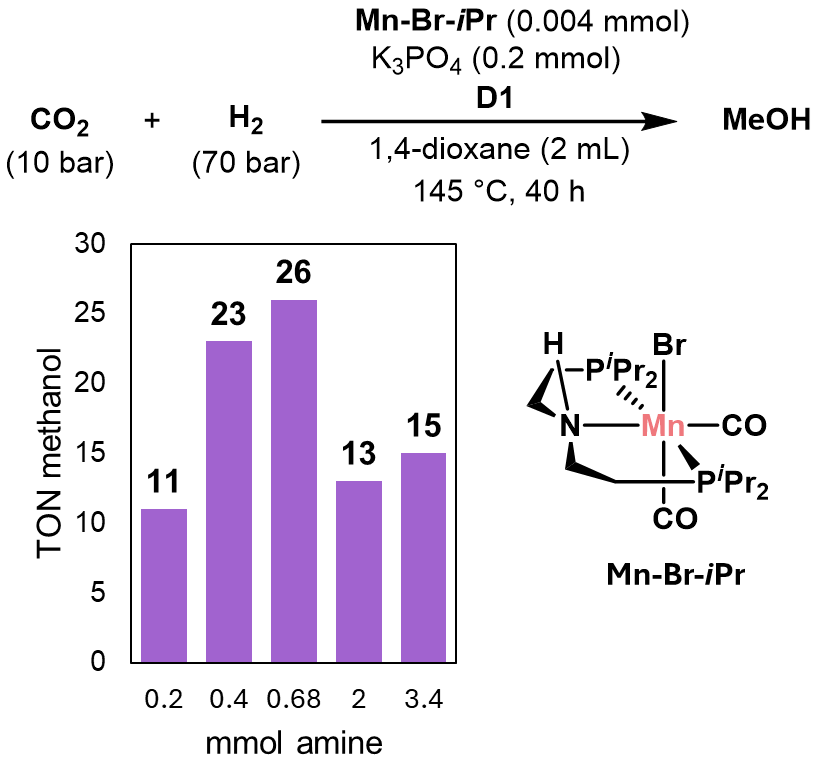


**Figure S1.** (a) CO_2_ hydrogenation to methanol catalyzed by **Mn-1-iPr** complex. (b) Optimization of the amount of **D1** (in mmol) added to the reaction.

The formation of methanol did not increase considerably when 0.4 and 0.68 mmol of amine were added. Therefore, the optimized amount of amine chosen was 0.4 mmol for a 2 mL solvent scale. K_3_PO_4_ was used as a base for the optimization of the amount of amine in glass vials and it performed similarly to KO*t*Bu. However, due to solubility issues, KO*t*Bu was employed for the scale-up process.

## **Catalytic testing**

In a glovebox, the corresponding catalyst (40 µmol) was weighed into a Schlenk tube equipped with a magnetic stir bar, and 1,4-dioxane (20 mL) was added, followed by KO*t*Bu (224.4 mg, 2 mmol). The reaction mixture was stirred for approximately 5 minutes. The amine **D1**, (430 µL, 4 mmol) or **M1** (2 mL of a 2 M solution of **M1** in THF, 4 mmol) was added, and the Schlenk tube was closed with a stopper and taken out of the glovebox. Using a syringe, the reaction mixture was transferred to a 300 mL autoclave (previously degassed and refilled with Ar three times) containing a magnetic stir bar, and the autoclave was carefully flushed ten times with 5-10 bar CO_2_ before being charged with 10 bar CO_2_ and 70 bar H_2_. The autoclave was placed in a preheated aluminium block at 145 ^o^C for 40 h. After the reaction time, the autoclave was cooled to room temperature in an ice bath and the gas pressure was carefully and slowly released over a period of 2 hours. Defined amounts of 1,3,5-trimethoxybenzene and hexadecane were added as the internal standards. The reaction mixture was filtered through Celite and analyzed in two different GCs.

**Table S1**. Effect of the phosphine substituents on methanol TON.^a^


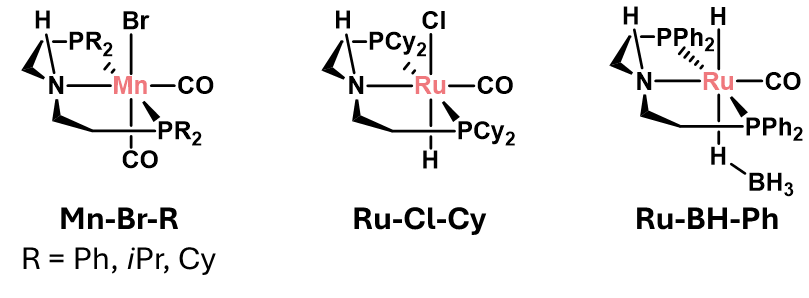


| **entry** | **catalyst** | **TON (CH_3_OH)^b^** |
| --- | --- | --- |
| 1 | **Ru-BH-Ph** | 178.9 |
| 2 | **Ru-Cl-Cy** | 48.5 |
| 3 | **Mn-Br-Ph** | 45.2 |
| 4 | **Mn-Br-*i*Pr** | 18.9 |
| 5 | **Mn-Br-Cy** | 14.0 |

^a^Reaction conditions: **D1** (4 mmol), KO*t*Bu (2 mmol), catalyst (40 µmol), CO_2_ (10 bar), H_2_ (70 bar), 1,4-dioxane as solvent (20 mL), 145 ^o^C for 40 h. ^b^Methanol TON were determined by GC analysis in two different instruments using 1,3,5-trimethoxybenzene and hexadecane as internal standards. Average of two runs.

# **Mechanistic study for CO_2_ hydrogenation to methanol**

Amine-assisted CO_2_ hydrogenation to methanol is a multistep sequential reaction, as illustrated in Scheme 1 in the manuscript. The current study focused on the role of amine in the hydrogenation process, so only the amine-dependent reaction mechanisms were initially considered. These mechanisms included the formation of Ru-carbamate complex and formamide hydrogenation (reactions *i* and *iv* in Scheme 1 of the manuscript)

## **Carbamate complex formation**

The Ru-formate complex (**Ru-3-Ph**) is the primary resting state for CO₂ hydrogenation to methanol, which arises from the reaction of CO₂ with the catalyst (**Ru-1-Ph**) with ΔG = -5.8 kcal/mol with DLPNO-CC methods (Figure S2). However, in the presence of amine, CO₂ can form carbamic acid, which then combines with the dehydrogenated catalyst **(Ru-5-Ph**) to generate the secondary resting state (**Ru-2-Ph**); the stability of **Ru-2-Ph** depends on the nature of the amine. Figure S2 illustrates the stability of **Ru-2-Ph** with **M1** and **D1**, resulting from the addition of one and two CO_2_, **Ru-2-Ph-M1**, **Ru-2-Ph-D1-m**, and **Ru-2-Ph-D1-d** (where **m** and **d** stand for monocarbamate and dicarbamate), in comparison to **Ru-3-Ph**. Interestingly, **Ru-2-Ph-M1** and **Ru-2-Ph-D1-d** have similar ΔG energies of −6.3 and -6.5 kcal/mol, which are slightly more negative than the formate complex (**Ru-3-Ph**, ΔG = −5.8 kcal/mol). The most stable intermediate is **Ru-2-Ph-D1-m** with a ΔG of -8.9 kcal/mol, which does not explain the significant activity observed in the case of **D1** compared to **M1**. All the mentioned ΔG values used **Ru-1-Ph**, CO_2_, and amine as a reference, utilizing DLPNO-CC method.


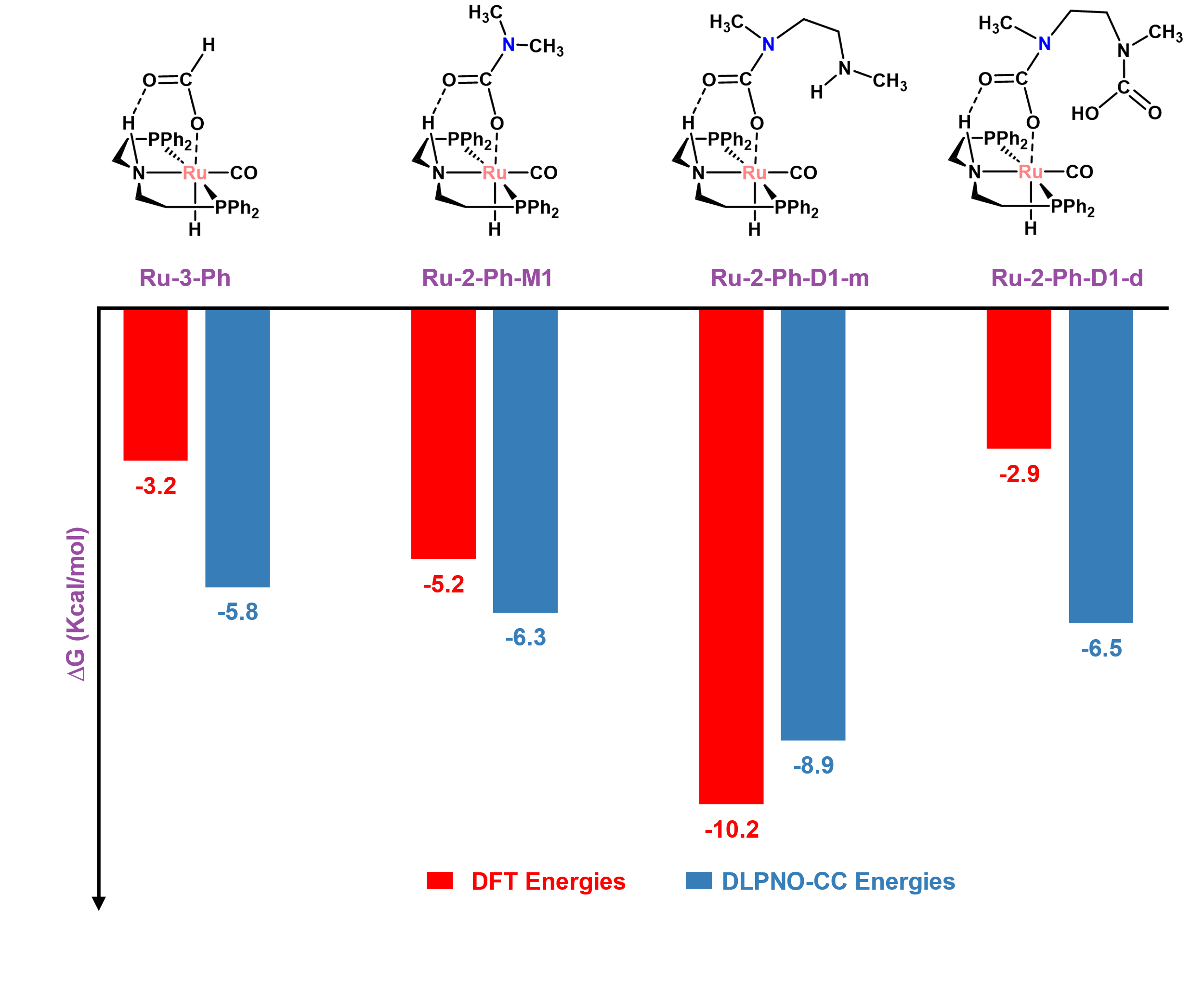


**Figure S2.** DFT and DLPNO-CC Gibbs free energies (in kcal/mol) for the proposed resting states in amine-assisted CO_2_ hydrogenation to methanol.

## **Formamide hydrogenation to formaldehyde**

The formamide hydrogenation to formaldehyde also depends on the amine used for the reaction. In a previous work[4], we showed that this step occurs through a metal-ligand cooperative mechanism, forming a hemiaminal. The hemiaminal is then hydrogenated via a methanol-assisted, catalyst-independent mechanism to recover amine and produce formaldehyde[4]. Subsequently, the Ru-MACHO-Ph hydrogenates formaldehyde to methanol in a barrierless reaction.

The energy profiles for the hydrogenation of dimethylformamide (DMF), derived from **M1,** and N-methylformamide, derived from **D1**, to their corresponding hemiaminals are shown in Figure S3. The hydrogenation of DMF to formaldehyde was reported in our earlier work, and the energies are given in parentheses [2]. Comparison of the results with the two amines shows slight differences in energy barriers: **TS1** is 1.8 kcal/mol lower, while **TS2** is 1.5 kcal/mol higher for N-methylformamide. Additionally, a difference of less than 1.0 kcal/mol was observed between the intermediates **Ru-4-Ph-D1-d** and **Ru-4-Ph-M1**, which is a key intermediate (the highest in energy) in the catalytic cycle of formamide hydrogenation. Although formamide hydrogenation does not proceed via the H₂-assisted mechanism, this mechanism is important because it helps form methanol at the reaction onset, and can then assist in the dissociation of hemiaminal. The energies were very close, with **TS3** being 0.7 kcal/mol higher for **D2**. Interestingly, the similar energies of the key intermediate and transition state failed to explain the higher methanol yield seen with **D2** compared to **M1**.

**Figure S3.** Gibbs free-energy profile (SMD-THF) for the hydrogenation of formamides derived from **M1** and **D1** to formaldehyde over **Ru-1-Ph**; values in parentheses refer to the formamide derived from the **M1** pathway. R = CH_3_ or CH_2_CH_2_NCH_3_CHO.

## **Comparison of Ru-4-Ph-D1-d and Ru-4-Ph-M1 using DLPNO-CC energies.**

**Scheme S1**. Comparison of the ΔG of formation for Ru**-4-Ph-M1** and **Ru-4-Ph-D1-d** using DLPNO-CC method.

# **Microkinetic modeling**

To gain a deeper understanding of the mechanisms involved in **D1**-assisted CO_2_ hydrogenation to methanol, we constructed a microkinetic model (MKM) that simulates the production of methanol using **D1** as a co-catalyst. The constructed model is then compared to our previously reported MKM for **M1**-assisted CO_2_ hydrogenation to methanol. The two microkinetic models were built using DLPNO-CC energies. The time-dependent concentrations of key species for M1-based and D1-based MKM are shown in Figures S4-S5 and Figures S6-S7, respectively. To mimic the experimental conditions of this study, 0.4 mol/L CO_2_, 0.144 mol/L of H_2_ (constant), 0.02 mol/L of catalyst, and 0.196 mol/L of **D1** were used in the microkinetic model.

The same elementary steps used for the **M1**-model were used for the **D1**-model, except for the two amidation steps needed for **D1** and N-methylformamide hydrogenation to hemiaminal (mechanisms in Figure S3), which were calculated. Additionally, hydrogenation of a single formamide group of N-methylformamide does not fully recover the **D1** amine but produces a **D1-formamide-m**. Consequently, three additional reactions were included to recover the **D1** from the **D1-formamide-m**. The ΔG_F_ and ΔG_B_ in Table S2 represent the Gibbs free-energy differences between the transition state and reactants, and between the transition state and products, respectively. The simulated methanol turnover numbers (TON) were 4.4 for **M1** and 198.5 for **D1**. These results align with the experimental TONs of **M1** and **D1**, which are 3.3 and 179.8, respectively.

**Table S2**. The reactions utilized for constructing the MKM and their corresponding ΔG_F_ and ΔG_B_ at the DLPNO-CCSD(T)/cc-pVTZ-DK//M06L/def2-SVP level of theory. The energies for the amine-independent reactions were obtained from our previous study[7].

| # | **Reaction** | ΔG_F_ | ΔG_B_ |
| --- | --- | --- | --- |
| **R01** | CO_2_ + Ru-1-Ph = Ru-3-Ph | 10.1 | 15.9 |
| **R02** | Ru-3-Ph = Ru-5-Ph + HCOOH | 30.0 | 4.4 |
| **R03** | HCOOH + D1 = D1-formamide-m + H_2_O | 18.8 | 28.7 |
| **R04** | HCOOH + D1-formamide-m = N-methylformamide + H_2_O | 18.2 | 27.7 |
| **R05** | Ru-5-Ph + H_2_ = Ru-1-Ph | 16.1 | 32.4 |
| **R06** | Ru-1-Ph + N-methylformamide = Ru-4-Ph-D1-d | 26.1 | 4.4 |
| **R07** | Ru-4-Ph-D1-d = Ru-5-Ph + D1-d-HA | 6.4 | 4.4 |
| **R08** | Ru-4-Ph-D1-d + H_2_ = Ru-1-Ph + HCHO + D1-formamide-m | 10.3 | 19.7 |
| **R09** | Ru-1-Ph + HCHO = Ru-5-Ph + CH_3_OH | 4.4 | 4.4 |
| **R10** | CH_3_OH + D1-d-HA = D1-formamide-m + HCHO + CH_3_OH | 16.7 | 13.7 |
| **R11** | 2CO_2_ + D1 = D1-carbemic acid-d | 9.7 | 4.4 |
| **R12** | Ru-5-Ph + D1-carbemic acid-d = Ru-2-Ph-D1-d | 4.4 | 30.6 |
| **R13** | Ru-3-Ph + H_2_ = Ru-1-Ph + FA | 13.8 | 4.4 |
| **R14** | Ru-5-Ph + H_2_ + CH_3_OH = Ru-1-Ph + CH_3_OH | 4.4 | 21.9 |
| **R15** | Ru-3-Ph + CH_3_OH = Ru-8-Ph + HCOOH | 21.5 | 4.4 |
| **R16** | Ru-8-Ph = Ru-5-Ph + CH_3_OH | 11.0 | 4.4 |
| **R17** | Ru-4-Ph-D1-d + CH_3_OH = Ru-8-Ph + HCHO + D1-formamide-m | 4.4 | 4.4 |
| **R18** | Ru-2-Ph-D1-d + H_2_ = D1-carbemic acid-d + Ru-1-Ph | 14.3 | 4.4 |
| **R19** | Ru-1-Ph + D1-formamide-m = Ru-4-Ph-D1-m | 25.3 | 4.4 |
| **R20** | Ru-4-Ph-D1-m = Ru-5-Ph + D1-m-HA | 4.4 | 5.3 |
| **R21** | D1-m-HA + CH_3_OH = D1 + HCHO + CH_3_OH | 18.4 | 9.3 |

**Table S3.** Comparison of energies calculated with the M06/def2-TZVP and DLPNO-CCSD(T)/cc-pVTZ-DK methods for key Ru species in the microkinetic model.

| **Species** | **DLPNO-CCSD(T)** | **M06** |
| --- | --- | --- |
| Ru**-4-Ph-M1** | 24.1 | 17.8 |
| Ru**-4-Ph-D1-d** | 23.6 | 17.1 |
| Ru**-3-Ph** | -5.8 | -3.2 |
| Ru**-2-Ph-M1** | -5.2 | -6.3 |
| Ru**-2-Ph-D1-d** | -2.9 | -6.5 |


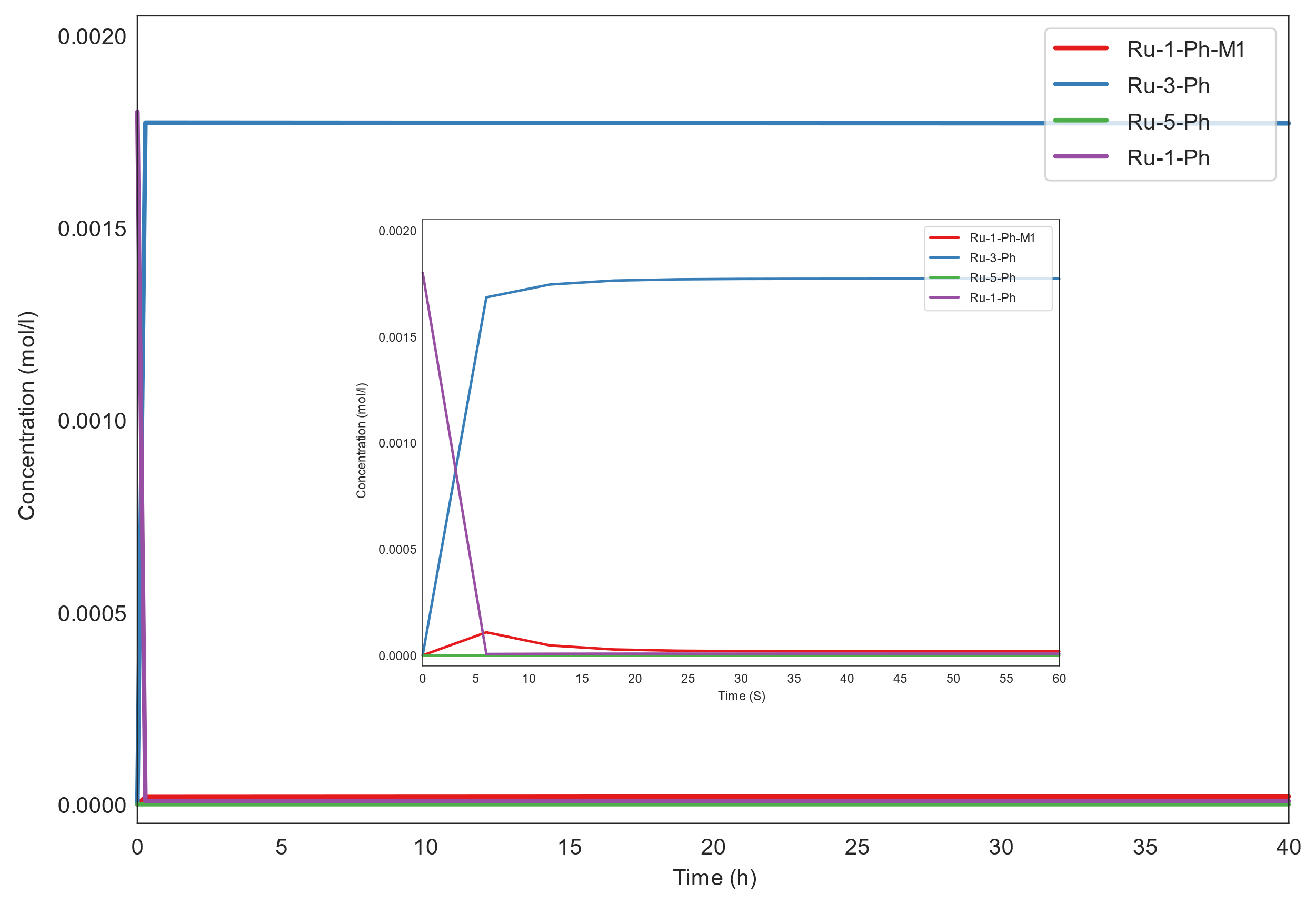


**Figure S4.** Time-dependent concentrations of key metal-complex species in M1-based microkinetic modeling.

**
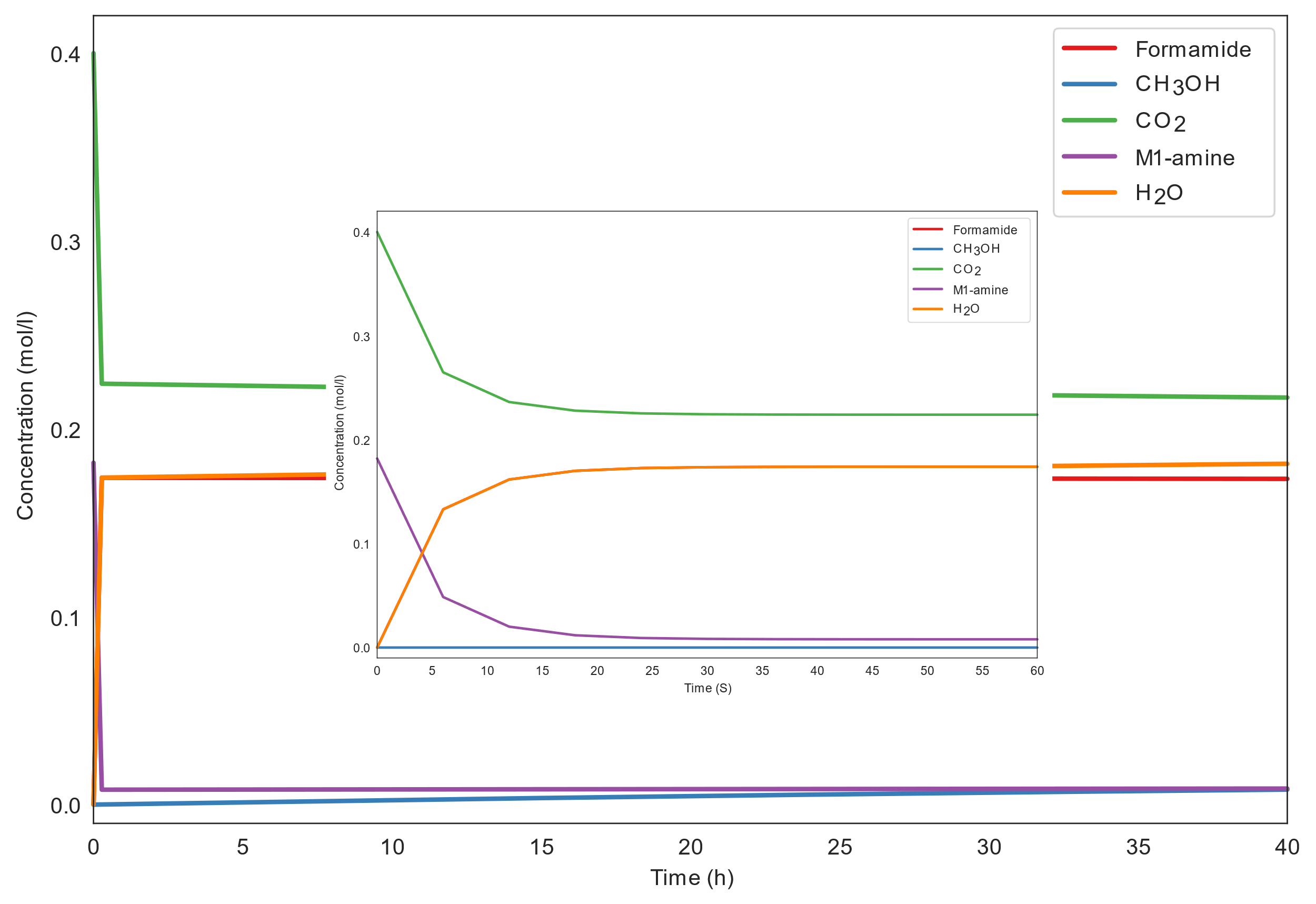
**

**Figure S5.** Time-dependent concentrations of key organic species in M1-based microkinetic modeling.

**
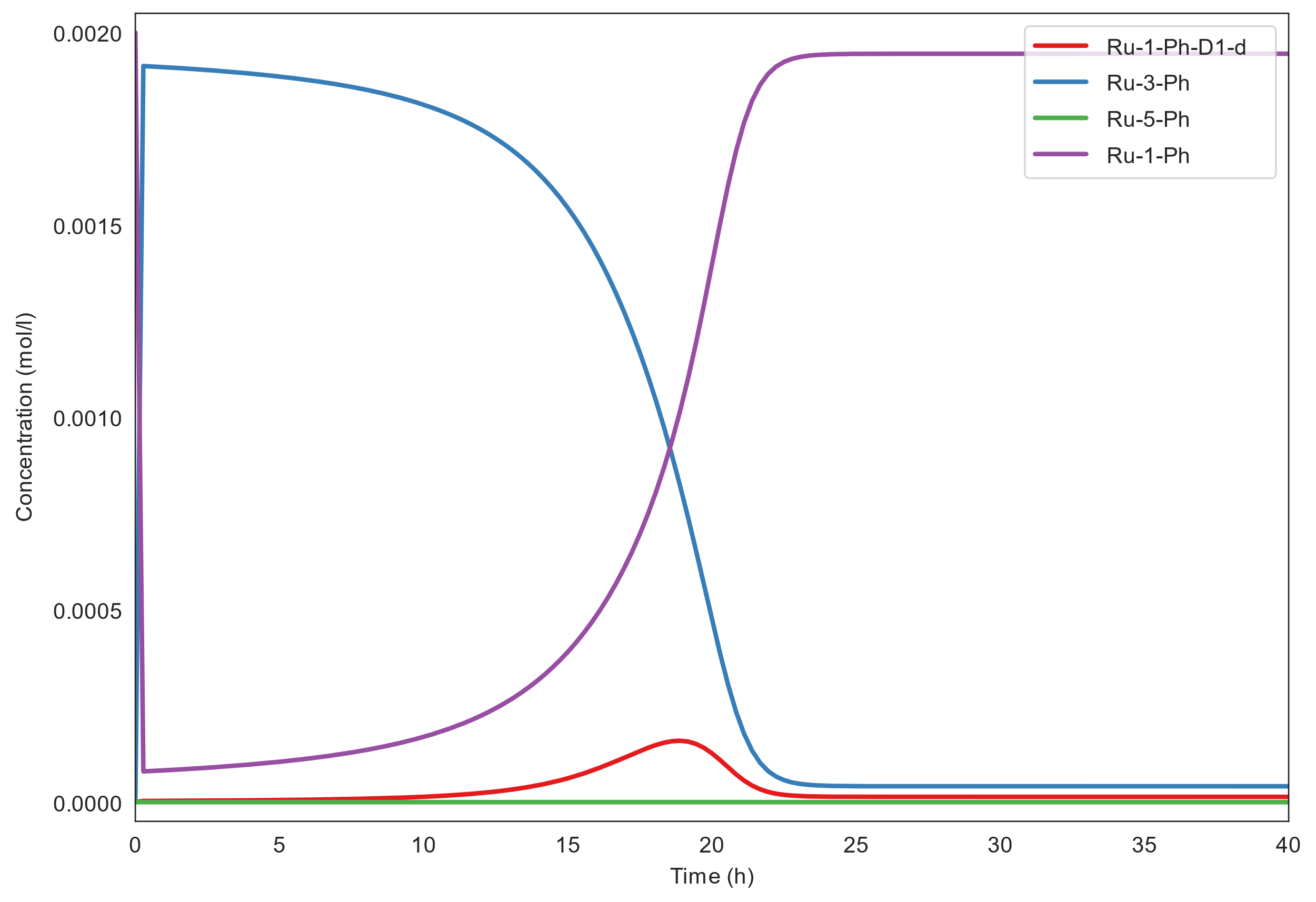
**

**Figure S6.** Time-dependent concentrations of key metal-complex species in D1-based microkinetic modeling.

**
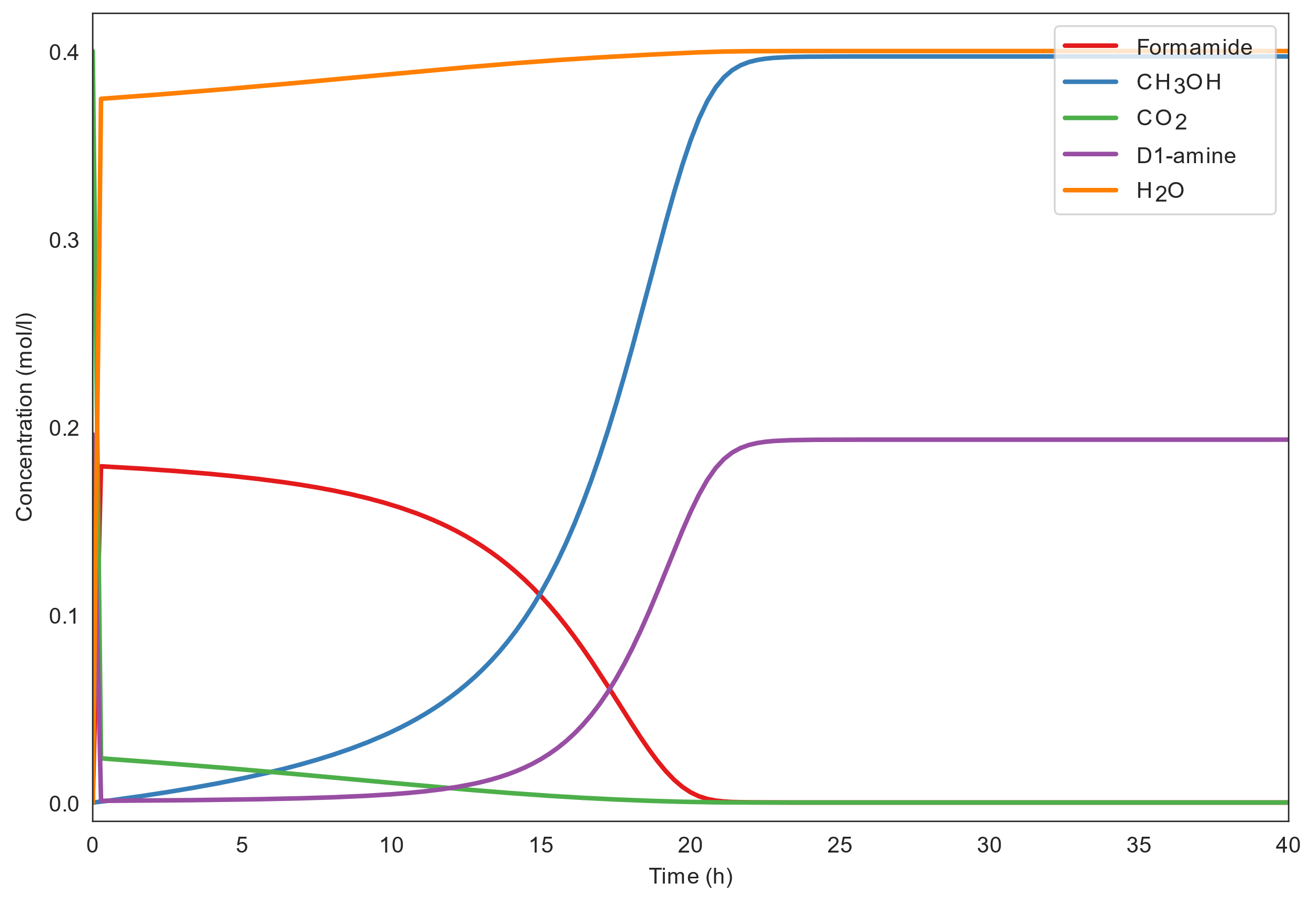
**

**Figure S7.** Time-dependent concentrations of key organic species in D1-based microkinetic modeling.

# **ΔG_amidation_ of the investigated amines**

The Gibbs free energy of formamide formation (ΔG_amidation_) was calculated for all amines depicted in Figure S8. The results, presented in Figure S9, reveal a clear trend in formamide stability where diamines form more stable formamides. Among monoamines (**M**), ΔG_amidation_ varied from -10.2 to 3.8 kcal/mol. **M14** and **M16** had ΔG_amidation_ > 0, probably because the N lone pair in these amines can be delocalized in aromatic rings. All secondary monoamines (**M1**-**M3**, **M8**, **M12** and **M15**) yielded more stable formamides compared to the primary amines. The only exception was **M9**, which forms a less stable formamide with ΔG_amidation_ of -6.7 kcal/mol. This result may be attributed to the steric hindrance between the two propyl groups and the formyl group of the formamide.

In the case of diamines, the ΔG_amidation_ corresponds to their reaction with two molecules of formic acid, yielding a double amide, unless they contain a tertiary amine, which cannot react with formic acid. The energies obtained vary from -6.6 to -19.4 kcal/mol. The least negative values correspond to **D2 (**ΔG_amidation_ = -6.6 kcal/mol**)** and **D5 (**ΔG_amidation_ = -8.7 kcal/mol**)**, both containing a tertiary amine. **D1** formed the most stable formamide among the diamines, as it contains two secondary amines. **D4**, with one secondary and one primary amine, ranks next in stability, highlighting the role of secondary amines in stabilizing formamides. Transitioning from monoamine to diamine involves a significant increase in ΔG_amidation_. Comparing **M2** (ΔG_amidation_ = -10.2 kcal/mol) with the most stable diamine **D1** (ΔG_amidation_ = -18.8 kcal/mol), the energy changes by 8.6 kcal/mol.

**Figure S8.** Molecular structures of the studied amines.

**Figure S9.** The ΔG_amidation_ for all amines in Figure S8, considering the addition of one molecule of formic acid for each primary and secondary amine. All energies were obtained using the DFT method.

**Sensitivity analysis of the ΔG_amidation_ in the formation of methanol.**

The microkinetic model constructed for **D1** was modified by merging the **R03** and **R04** reactions, meaning the amidation reaction happens in one single step to enable us to scan the ΔG_amidation_. Figure **S10** illustrates how the amount of produced methanol changes with the variation in the value of ΔG_amidation_. Increasing the energy for the formamide formation (less negative) decreases the methanol TON until suppressing it at energies > 8.0 kcal/mol. Increasing ΔG_amidation_ of **D1** (originally was -18.8 kcal/mol) by more than 8.0 kcal/mol means moving ΔG_amidation_ to the range of monoamines, explaining why no or little methanol is formed in these cases. On the other hand, decreasing ΔG_amidation_ increases methanol formation, but not by a significant difference, which is in agreement with the experimental results.

**
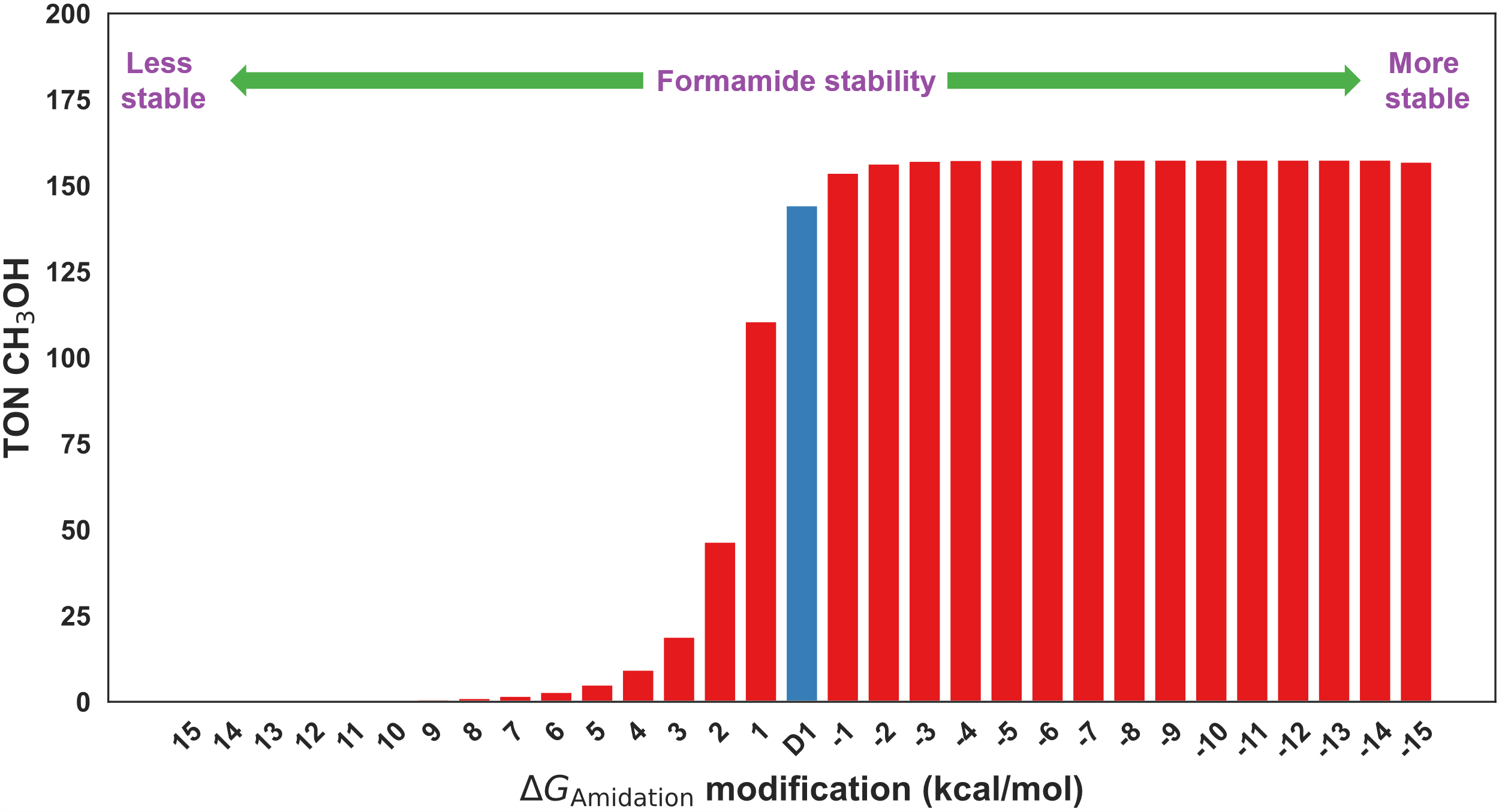
**

**Figure S10.** Simulated methanol TON as a function of ΔG_amidation._ The highlighted blue bar marks the ΔG_amidation_ value for **D1.**

# **Role of amine in Ru-3-Ph activation**

The energy profiles in Figure S11 qualitatively explain the correlation between the amidation reaction and the methanol TON. In the case of **M1**, in red, formate is the catalyst resting state, while the amide hydrogenation is the rate-limiting step of the reaction. By making the amidation reaction more exergonic, using **D1** instead of **M1**, the equilibrium between **Ru-3-Ph** and **Ru-1-Ph** + amide is displaced towards **Ru-1-Ph**. As the amide hydrogenation energy barrier is almost the same for both amines, the energy span of the reaction decreases for **D2**, favoring the formation of methanol. Once the equilibrium is entirely displaced, the amidation reaction is expected to have little influence on the methanol formation, as suggested by Figure S10.

**Figure S11.** Schematic representation of the energy profiles for the formic acid formation and amide hydrogenation steps with **M1** and **D1**, to show the impact of the amidation reaction in the equilibrium between **Ru-3-Ph** and **Ru-1-Ph** and in the formation of methanol. All the energies here were calculated using CC methods.

**Figure S12.** Schematic representation of the energy profiles for the formic acid formation and amide hydrogenation steps with **M1** and **D1**, to show the impact of the amidation reaction in the equilibrium between **Mn-3-Ph** and **Mn-1-Ph** and in the formation of methanol. All the energies here were calculated using CC methods.

# **Linear fitting for the correlation between the ΔG amidation and methanol formation**


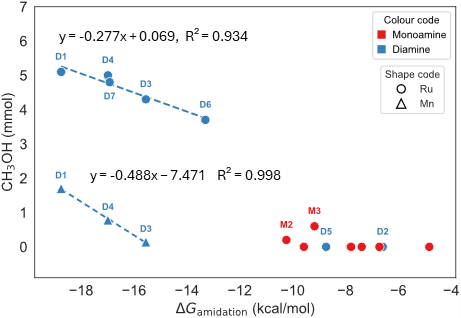


**Figure S13.** Correlation between the DFT Gibbs free energy of formamide formation (ΔG_amidation_) and the amount of methanol produced (mmol) for the investigated amines. Experimental conditions: CO₂/H₂ = 1:3 (75 bar) at 145 °C for 20 h in triglyme (10 ml), with the total amine functionality held constant at 30.6 mmol[8] for Ru-BH-Ph (10 mmol) (Figure 3a), and CO₂/H₂ = 1:7 (80 bar) at 145 °C for 40 h in dioxane (20 ml) with 4 mmol of amine Mn-Br-Ph (40 mmol). See Figure S8 for amine labels

# **Cartesian coordinates of the investigated amines**

**Table S4.** Cartesian coordinates of the investigated amines and their corresponding formamides.

| **#** | **Amine** | **Formamide** |
| --- | --- | --- |
| M1 | 10  M1 Energy: -84784.4336812  N 0.00000 0.55963 -0.15163  H -0.00000 1.32579 0.51878  C 1.20218 -0.21775 0.01991  H 1.27633 -0.98161 -0.77083  H 2.09059 0.42368 -0.06684  H 1.27348 -0.75717 0.98951  C -1.20218 -0.21775 0.01991  H -1.27348 -0.75717 0.98951  H -2.09059 0.42368 -0.06684  H -1.27633 -0.98161 -0.77084 | 12  M1 Energy: -155907.9712059  N 0.34401 -0.01823 -0.00016  C 1.57548 -0.76201 0.00006  H 2.18273 -0.53318 0.89033  H 1.35991 -1.83798 -0.00079  H 2.18367 -0.53197 -0.88924  C 0.42988 1.41973 0.00001  H 0.96489 1.78551 -0.89037  H -0.58413 1.83362 0.00045  H 0.96555 1.78521 0.89012  C -0.86233 -0.63957 -0.00003  H -0.74191 -1.75072 -0.00010  O -1.94962 -0.09147 0.00006 |
| M2 | 28  M2 Energy: -232741.5057014  N -0.00382 -0.17979 0.96018  C -0.92990 -0.62909 -0.05794  H -0.60072 -0.23520 -1.03798  H -0.93158 -1.73823 -0.17255  C 1.35195 -0.66315 0.79766  H 1.40033 -1.77228 0.69297  H 1.90737 -0.42307 1.72158  C -2.35354 -0.14953 0.20240  H -2.65253 -0.54063 1.19532  C 2.09278 -0.04084 -0.38583  H 1.59554 -0.37973 -1.31461  C -3.30582 -0.72316 -0.83260  H -3.28028 -1.82299 -0.85250  H -4.34569 -0.42176 -0.63919  H -3.04971 -0.37229 -1.84553  C -2.43474 1.36748 0.25137  H -2.14369 1.80811 -0.71666  H -3.45591 1.71221 0.47255  H -1.76500 1.78728 1.01460  C 2.03789 1.47653 -0.35283  H 2.58016 1.91869 -1.20212  H 1.00459 1.85050 -0.38278  H 2.49739 1.86947 0.56924  C 3.52425 -0.54856 -0.42310  H 4.08587 -0.22284 0.46772  H 3.57003 -1.64780 -0.45267  H 4.06672 -0.17435 -1.30360  H -0.35340 -0.47459 1.87019 | 30  M2 Energy: -303866.1540422  N 0.00551 0.72180 0.12728  C -0.93385 -0.00497 -0.70737  H -1.58267 0.75138 -1.17363  H -0.38812 -0.50594 -1.52622  C 1.06476 -0.00661 0.79685  H 0.62980 -0.75293 1.48562  H 1.60509 0.71543 1.43178  C -1.78517 -1.02540 0.04663  H -1.10912 -1.79662 0.46243  C 2.05432 -0.69206 -0.14447  H 1.49917 -1.45218 -0.72591  C -2.72779 -1.71342 -0.92578  H -2.18520 -2.19375 -1.75360  H -3.32445 -2.49143 -0.42836  H -3.43388 -0.99427 -1.37085  C -2.53761 -0.38054 1.19787  H -3.21508 0.40960 0.83569  H -3.15035 -1.11548 1.74003  H -1.85928 0.08264 1.92968  C 2.67109 0.29699 -1.11793  H 3.21269 1.09466 -0.58316  H 3.39171 -0.19404 -1.78785  H 1.91555 0.78424 -1.75156  C 3.11222 -1.41540 0.67091  H 3.81060 -1.96802 0.02646  H 3.70967 -0.70727 1.26734  H 2.66824 -2.13891 1.37074  C -0.11514 2.06703 0.27670  H 0.68604 2.47191 0.94371  O -0.96701 2.77433 -0.23046 |
| M3 | 17  M3 Energy: -158011.0863185  C -1.25280 0.70326 -0.22659  C -1.20662 -0.75161 0.22441  C 1.20665 -0.75157 0.22442  C 1.25278 0.70330 -0.22659  C -0.00002 1.44383 0.21947  H -1.26217 -0.78978 1.32844  H -2.08452 -1.30585 -0.14302  H -1.32703 0.73486 -1.32920  H -2.16128 1.19650 0.15578  H 1.26219 -0.78973 1.32844  H 2.08456 -1.30578 -0.14302  H 2.16124 1.19657 0.15578  H 1.32701 0.73490 -1.32920  H -0.00003 1.52125 1.32247  H -0.00004 2.47970 -0.15549  N 0.00002 -1.46000 -0.18298  H 0.00003 -1.53589 -1.20081 | 19  M3 Energy: -229134.9088015  C 1.08085 -1.38807 -0.26901  C -0.16644 -1.08395 0.54357  C 0.30833 1.33346 0.42046  C 1.56247 1.06883 -0.39571  C 2.13390 -0.30582 -0.07965  H -0.98587 -1.78164 0.32903  H 0.05929 -1.16002 1.62363  H 1.47272 -2.37597 0.01647  H 0.80616 -1.45628 -1.33570  H -0.17819 2.27580 0.12912  H 0.57210 1.42522 1.49105  H 1.31078 1.12888 -1.46863  H 2.30192 1.86021 -0.20110  H 3.01844 -0.51069 -0.70172  H 2.48675 -0.32043 0.96755  N -0.65114 0.25650 0.25927  C -1.91965 0.47316 -0.16443  H -2.10232 1.56063 -0.35147  O -2.77506 -0.37837 -0.33056 |
| M4 | 16  M4 Energy: -134108.7736674  C -2.51307 -0.31428 0.00001  H -3.41669 0.31212 -0.00003  H -2.57075 -0.96731 0.88457  H -2.57074 -0.96740 -0.88448  C -1.24540 0.51315 -0.00002  H -1.23910 1.18515 0.87649  H -1.23913 1.18512 -0.87655  C 0.02559 -0.31814 -0.00002  H 0.03078 -0.98932 -0.87923  H 0.03075 -0.98938 0.87914  C 1.30374 0.50637 0.00003  H 1.30243 1.17870 0.87626  H 1.30247 1.17877 -0.87614  N 2.54868 -0.24373 0.00002  H 2.55194 -0.87162 0.80451  H 2.55210 -0.87131 -0.80471 | 18  M4 Energy: -205230.4059140  C -2.94051 -0.89900 -0.13518  H -3.70125 -0.22982 0.29546  H -3.18272 -1.92010 0.19182  H -3.06823 -0.86909 -1.22821  C -1.54137 -0.49021 0.27225  H -0.81032 -1.20537 -0.14341  H -1.43129 -0.56779 1.36880  C -1.17402 0.91700 -0.16550  H -1.89251 1.64064 0.25518  H -1.27620 1.00598 -1.26209  C 0.22578 1.34544 0.24157  H 0.34380 1.26862 1.33454  H 0.38899 2.40502 -0.01113  N 1.27323 0.54565 -0.35436  H 1.42099 0.61539 -1.35905  C 2.01969 -0.36581 0.30718  H 1.76051 -0.38760 1.39755  O 2.87477 -1.07751 -0.18511 |
| M5 | 16  M5 Energy: -134110.2628472  C 1.73117 -0.75583 0.00777  H 2.62122 -0.29989 -0.45045  H 1.90072 -0.77280 1.09682  C 0.46196 0.00400 -0.33642  H 0.34017 -0.00829 -1.43774  C -0.76298 -0.70142 0.25553  H -0.65083 -0.74223 1.35477  H -0.75586 -1.75455 -0.07652  N -2.06467 -0.13450 -0.04643  H -2.11775 0.81369 0.32404  H -2.15503 -0.02270 -1.05677  H 1.68721 -1.80231 -0.33010  C 0.54748 1.45430 0.10820  H 1.40948 1.96899 -0.34130  H -0.34999 2.02650 -0.17114  H 0.65756 1.52884 1.20291 | 18  M5 Energy: -205231.8394462  C -1.09618 1.48921 -0.01153  H -1.07731 1.64096 1.08029  H -0.12970 1.83316 -0.40818  C -1.37466 0.03689 -0.35773  H -1.37453 -0.06213 -1.46009  C -0.28064 -0.88762 0.17744  H -0.54073 -1.93727 -0.03716  H -0.23017 -0.80288 1.27637  N 1.03764 -0.60646 -0.33905  H 1.22211 -0.79884 -1.32143  C 2.02766 -0.00476 0.35733  H 1.71712 0.19384 1.41592  O 3.12633 0.28618 -0.07622  H -1.87013 2.15533 -0.41948  C -2.72966 -0.41298 0.15974  H -3.54270 0.20779 -0.24284  H -2.94759 -1.45701 -0.11001  H -2.77956 -0.34159 1.25824 |
| M6 | 16  M6 Energy: -134111.1568781  C -2.07002 -0.02116 -0.11215  H -2.22474 -0.04730 -1.20200  H -2.92514 -0.53457 0.35038  C -0.75329 -0.65821 0.27473  H -0.74608 -1.72131 -0.01859  C 0.47405 0.02017 -0.32610  H 0.34507 0.03188 -1.42572  N 0.52035 1.41898 0.10115  H 0.71173 1.43880 1.10512  H 1.33570 1.86552 -0.31843  H -2.10872 1.03394 0.19243  H -0.64370 -0.65992 1.37556  C 1.74395 -0.75342 -0.00629  H 2.62917 -0.27134 -0.44779  H 1.90670 -0.80552 1.08281  H 1.70940 -1.78733 -0.38299 | 18  M6 Energy: -205232.5238645  C -1.12287 -1.97935 0.05817  H -1.83549 -2.73791 -0.29414  H -0.16135 -2.18636 -0.43501  C -1.61318 -0.57764 -0.22761  H -2.60443 -0.42115 0.22862  C -0.68055 0.52129 0.27937  H -0.52865 0.37107 1.36367  N 0.64411 0.40927 -0.31419  H 0.73865 0.61563 -1.30736  C 1.74728 -0.03865 0.32313  H 1.53142 -0.26100 1.40030  O 2.84855 -0.18363 -0.17420  H -0.97710 -2.14529 1.13693  H -1.76319 -0.43816 -1.31321  C -1.24759 1.90762 0.04574  H -1.38009 2.10284 -1.02974  H -2.23099 2.01904 0.52262  H -0.58445 2.68577 0.44748 |
| M7 | 16  M7 Energy: -134114.5096367  C -0.00000 -0.00767 0.01772  C 0.00013 1.27815 -0.81053  H -0.88798 1.89080 -0.58927  H 0.88827 1.89070 -0.58916  H 0.00019 1.07717 -1.89352  C -1.24906 -0.82212 -0.29455  H -1.30048 -1.09033 -1.35993  H -1.27005 -1.75181 0.29282  H -2.16258 -0.25642 -0.05305  C 1.24897 -0.82229 -0.29444  H 1.30047 -1.09048 -1.35982  H 2.16255 -0.25672 -0.05283  H 1.26977 -1.75200 0.29291  N -0.00005 0.26690 1.46362  H 0.80536 0.85715 1.67870  H -0.80546 0.85715 1.67864 | 18  M7 Energy: -205233.0436451  C 0.78469 0.00749 0.00000  C 1.69788 1.22481 -0.00015  H 2.75201 0.91668 -0.00011  H 1.53113 1.84891 -0.89053  H 1.53113 1.84913 0.89008  C 1.03038 -0.82817 -1.25363  H 0.84322 -0.24081 -2.16409  H 2.07113 -1.18159 -1.28637  H 0.38197 -1.71554 -1.28550  C 1.03038 -0.82788 1.25383  H 0.38194 -1.71521 1.28592  H 2.07112 -1.18133 1.28665  H 0.84325 -0.24029 2.16415  N -0.60018 0.52149 -0.00005  H -0.73489 1.52990 -0.00002  C -1.72315 -0.22536 -0.00003  H -1.50507 -1.32263 0.00002  O -2.86084 0.21213 0.00000 |
| M8 | 28  M8 Energy: -232738.6481971  N -0.00000 -0.66517 -0.18127  H 0.00002 -0.51697 -1.19149  C 1.21163 -1.36882 0.18735  H 1.22123 -1.47642 1.28797  H 1.23386 -2.41039 -0.20705  C 2.45918 -0.63418 -0.26306  H 2.45706 -0.56171 -1.36702  H 3.34061 -1.25043 -0.01664  C 2.62072 0.75376 0.33421  H 1.73086 1.35669 0.08954  H 2.61952 0.67368 1.43585  C 3.87398 1.46474 -0.12889  H 4.78306 0.90226 0.13570  H 3.88555 1.59466 -1.22233  H 3.97071 2.46530 0.31660  C -1.21164 -1.36884 0.18731  H -1.22126 -1.47647 1.28793  H -1.23386 -2.41039 -0.20713  C -2.45918 -0.63417 -0.26309  H -3.34061 -1.25045 -0.01677  H -2.45700 -0.56161 -1.36705  C -2.62076 0.75372 0.33428  H -1.73085 1.35664 0.08978  H -2.61970 0.67353 1.43592  C -3.87394 1.46477 -0.12891  H -3.97072 2.46528 0.31669  H -3.88535 1.59483 -1.22234  H -4.78307 0.90228 0.13549 | 30  M8 Energy: -303863.2875921  N 0.49956 -1.31971 0.37307  C -0.81935 -0.90600 0.82746  H -1.47794 -1.78942 0.79995  H -0.73231 -0.60503 1.88375  C -1.44438 0.20740 0.00247  H -1.47425 -0.11107 -1.05350  H -0.79998 1.10335 0.03024  C -2.84263 0.56968 0.47035  H -3.48040 -0.33103 0.43846  H -2.80729 0.86139 1.53507  C -3.47739 1.67668 -0.34353  H -3.56096 1.39975 -1.40559  H -4.48929 1.92067 0.00987  H -2.88458 2.60334 -0.29907  C 1.68251 -0.82585 1.05490  H 2.54312 -1.38969 0.66092  H 1.60868 -1.07833 2.12627  C 1.91685 0.66545 0.88550  H 2.79352 0.94470 1.49396  H 1.07216 1.22445 1.32710  C 2.12804 1.09617 -0.55543  H 1.28221 0.74257 -1.17108  H 3.01535 0.57826 -0.96109  C 2.28174 2.59301 -0.71652  H 1.38520 3.12968 -0.36849  H 3.13300 2.98029 -0.13563  H 2.44520 2.88045 -1.76480  C 0.59140 -2.15817 -0.68725  H 1.65591 -2.39812 -0.93452  O -0.35312 -2.61731 -1.30638 |
| M9 | 22  M9 Energy: -183423.6590319  N -0.01575 -0.54551 0.32481  H -0.07131 -0.30372 1.31445  C -1.26904 -0.18227 -0.32837  H -1.19846 -0.55889 -1.36738  C 1.21953 -0.00032 -0.24662  H 0.98733 0.30374 -1.28460  C -1.55199 1.31454 -0.40104  H -1.62118 1.75302 0.60817  H -0.76803 1.85699 -0.94964  H -2.50386 1.51927 -0.91473  C -2.40349 -0.91699 0.35997  H -2.21929 -2.00017 0.38214  H -2.51303 -0.57859 1.40331  H -3.36577 -0.74141 -0.14131  C 1.73509 1.21875 0.50451  H 2.65439 1.62088 0.05271  H 0.99656 2.03210 0.53614  H 1.97811 0.95413 1.54676  C 2.28298 -1.08622 -0.30724  H 2.53138 -1.44332 0.70486  H 1.92999 -1.95380 -0.88247  H 3.21492 -0.72654 -0.76934 | 24  M9 Energy: -254546.5867636  N -0.01044 0.31266 -0.00027  C -1.38842 -0.19440 -0.00027  H -2.00655 0.71574 -0.00127  C 1.11542 -0.63900 0.00034  H 0.64652 -1.63583 0.00073  C -1.68948 -0.98106 1.26324  H -1.50091 -0.38121 2.16477  H -2.74438 -1.28932 1.28203  H -1.08554 -1.89920 1.33457  C -1.68874 -0.98337 -1.26251  H -1.49948 -0.38523 -2.16504  H -1.08496 -1.90177 -1.33175  H -2.74369 -1.29144 -1.28141  C 1.95049 -0.52684 -1.26376  H 2.71936 -1.31193 -1.29280  H 1.32932 -0.63041 -2.16443  H 2.47089 0.44057 -1.32444  C 1.95015 -0.52569 1.26455  H 2.47071 0.44168 1.32443  H 1.32872 -0.62829 2.16515  H 2.71889 -1.31088 1.29458  C 0.21648 1.64744 -0.00051  H 1.30786 1.88527 -0.00068  O -0.63113 2.52639 -0.00113 |
| M10 | 40  M10 Energy: -331376.6119043  C -7.54661 -0.12622 0.19357  H -8.42509 0.49756 -0.02538  H -7.65711 -1.05704 -0.38406  H -7.60107 -0.40187 1.25815  C -6.24863 0.58114 -0.13284  H -6.24401 0.88054 -1.19596  H -6.18784 1.52830 0.43205  C -5.01210 -0.25256 0.15606  H -5.06936 -1.20073 -0.40994  H -5.01431 -0.55424 1.21985  C -3.70487 0.44845 -0.16619  H -3.64733 1.39548 0.40146  H -3.70387 0.75170 -1.22939  C -2.46950 -0.38604 0.12024  H -2.52586 -1.33199 -0.44924  H -2.47166 -0.69140 1.18283  C -1.16187 0.31603 -0.19864  H -1.10447 1.26085 0.37266  H -1.16010 0.62343 -1.26062  C 0.07297 -0.51997 0.08553  H 0.01754 -1.46298 -0.48885  H 0.06892 -0.83080 1.14653  C 1.38111 0.18376 -0.22734  H 1.43768 1.12556 0.34906  H 1.38494 0.49669 -1.28769  C 2.61550 -0.65350 0.05505  H 2.56248 -1.59326 -0.52476  H 2.60928 -0.97035 1.11424  C 3.92381 0.05375 -0.24896  H 3.96870 0.99352 0.33149  H 3.93588 0.36807 -1.30940  C 5.15853 -0.78005 0.04329  H 5.11258 -1.72129 -0.53269  H 5.14987 -1.08639 1.10618  C 6.47530 -0.07550 -0.25969  H 6.52253 0.15945 -1.33752  H 7.30834 -0.77504 -0.07756  N 6.74021 1.15081 0.47696  H 6.01617 1.83643 0.26366  H 6.63385 0.96832 1.47561 | 42  M10 Energy: -402498.3026084  C -7.86925 0.67331 0.46237  H -8.78963 0.75432 -0.13359  H -8.04120 -0.10595 1.22103  H -7.74742 1.62482 1.00285  C -6.66058 0.36032 -0.39367  H -6.83172 -0.57708 -0.95216  H -6.54085 1.13806 -1.16871  C -5.36909 0.24129 0.39715  H -5.48596 -0.53690 1.17402  H -5.19431 1.17919 0.95613  C -4.15120 -0.07353 -0.45239  H -4.03541 0.70419 -1.22962  H -4.32634 -1.01181 -1.01042  C -2.86022 -0.19140 0.33752  H -2.97669 -0.96715 1.11668  H -2.68321 0.74791 0.89311  C -1.64341 -0.51048 -0.51218  H -1.52748 0.26412 -1.29242  H -1.81990 -1.45071 -1.06642  C -0.35195 -0.62645 0.27709  H -0.46892 -1.39780 1.06053  H -0.17258 0.31566 0.82716  C 0.86298 -0.95224 -0.57247  H 0.98062 -0.18109 -1.35587  H 0.68475 -1.89458 -1.12238  C 2.15527 -1.06529 0.21587  H 2.04433 -1.83647 1.00084  H 2.32445 -0.12020 0.76385  C 3.36569 -1.38846 -0.64321  H 3.49419 -0.59609 -1.40285  H 3.16762 -2.30834 -1.21912  C 4.66305 -1.56209 0.12993  H 5.47126 -1.84531 -0.56553  H 4.57395 -2.40396 0.83711  C 5.09998 -0.33485 0.91570  H 6.04228 -0.54653 1.44675  H 4.36401 -0.08501 1.69604  N 5.25827 0.84262 0.09138  H 5.96227 0.82437 -0.64390  C 4.48500 1.94848 0.16245  H 3.72750 1.86292 0.98427  O 4.57986 2.92994 -0.55025 |
| M11 | 15  M11 Energy: -180546.5523353  C -1.16506 -0.74935 -0.19355  C -1.19450 0.71216 0.19320  C 1.19449 0.71217 0.19320  C 1.16506 -0.74935 -0.19355  H -2.09090 1.19189 -0.22867  H -1.27833 0.78127 1.30091  H -2.02601 -1.28927 0.22548  H -1.21429 -0.83910 -1.29867  H 2.09090 1.19189 -0.22866  H 1.27833 0.78128 1.30092  H 1.21429 -0.83909 -1.29868  H 2.02602 -1.28926 0.22547  O 0.00001 -1.38633 0.29238  N -0.00000 1.35783 -0.32359  H -0.00000 2.34240 -0.06780 | 17  M11 Energy: -251668.6281583  C 1.59476 0.96887 -0.37793  C 0.34052 1.31728 0.39704  C -0.14229 -1.08147 0.51328  C 1.13686 -1.31355 -0.26310  H 0.58946 1.44306 1.46629  H -0.09530 2.26179 0.04173  H 1.37747 0.99052 -1.46535  H 2.39077 1.69951 -0.17910  H 0.05022 -1.19185 1.59535  H -0.92457 -1.79782 0.23201  H 1.59637 -2.26992 0.02167  H 0.91162 -1.35640 -1.34797  O 2.08773 -0.30023 -0.00423  N -0.63496 0.25652 0.24243  C -1.91309 0.48038 -0.15015  H -2.09664 1.56920 -0.32487  O -2.76963 -0.37137 -0.30222 |
| M12 | 14  M12 Energy: -133347.0657149  C 0.76939 1.01725 -0.06946  C -0.76931 1.01718 -0.07023  C -1.14429 -0.44947 0.19597  C 1.14435 -0.44976 0.19534  H 1.16254 1.34675 -1.04349  H 1.19706 1.69753 0.68186  H -1.16143 1.34510 -1.04522  H -1.19791 1.69853 0.67959  H -1.31606 -0.60549 1.27559  H -2.05892 -0.77509 -0.31906  H 1.31722 -0.60651 1.27468  H 2.05843 -0.77516 -0.32081  N -0.00019 -1.26080 -0.20914  H -0.00051 -1.31123 -1.22887 | 16  M12 Energy: -204471.6324637  C -1.89392 0.48107 0.30155  C -1.63489 -0.93425 -0.19840  C -0.14674 -1.12967 0.04821  C -0.65710 1.23367 -0.15790  H -2.82537 0.92078 -0.07870  H -1.95072 0.49039 1.40200  H -1.84875 -0.99603 -1.27748  H -2.25036 -1.69646 0.29732  H 0.05894 -1.54292 1.05151  H 0.34138 -1.79995 -0.67503  H -0.41914 2.11486 0.45504  H -0.76745 1.57851 -1.20215  N 0.39201 0.22352 -0.05349  C 1.71476 0.47690 -0.01949  H 1.93415 1.57142 -0.09297  O 2.58632 -0.37145 0.08139 |
| M13 | 17  M13 Energy: -205059.2341835  C 2.30135 0.00013 0.22428  C 1.61253 1.20602 0.08635  C 0.24489 1.20307 -0.18653  C -0.46039 -0.00012 -0.32716  C 0.24507 -1.20318 -0.18639  C 1.61272 -1.20588 0.08650  H 3.37377 0.00023 0.43474  H 2.14573 2.15509 0.18714  H -0.28995 2.15216 -0.29800  H -0.28962 -2.15236 -0.29774  H 2.14607 -2.15485 0.18742  C -1.95206 -0.00024 -0.54773  H -2.23535 -0.88120 -1.14543  H -2.23543 0.88035 -1.14592  N -2.75315 0.00011 0.67202  H -2.48420 0.80575 1.23839  H -2.48362 -0.80472 1.23925 | 19  M13 Energy: -276179.5629631  C 3.01279 0.76223 -0.00834  C 1.85000 1.53166 0.02197  C 0.59891 0.91474 0.04470  C 0.49133 -0.48052 0.03635  C 1.66502 -1.24442 -0.00087  C 2.91579 -0.63005 -0.01912  H 3.99254 1.24566 -0.02838  H 1.91545 2.62271 0.02705  H -0.30824 1.52515 0.06624  H 1.59382 -2.33633 -0.01724  H 3.82088 -1.24207 -0.04890  C -0.84937 -1.16890 0.09999  H -1.09438 -1.40553 1.14945  H -0.78759 -2.14370 -0.41023  N -1.94677 -0.39847 -0.42372  H -1.95297 -0.17800 -1.41754  C -2.95675 0.10418 0.32406  H -2.83929 -0.17415 1.40309  O -3.88488 0.76775 -0.09373 |
| M14 | 27  M14 Energy: -349987.9044541  N 0.65475 1.60746 0.14178  H 0.93221 2.46255 0.60520  C 1.61623 0.63482 0.01732  C 2.84833 0.77062 0.69372  C 1.41046 -0.52253 -0.76266  C 3.82785 -0.20907 0.59085  H 3.02224 1.66419 1.30038  C 2.40437 -1.49548 -0.85458  H 0.46761 -0.66432 -1.29564  C 3.61896 -1.35483 -0.18333  H 4.77273 -0.07411 1.12460  H 2.21813 -2.38310 -1.46560  H 4.39099 -2.12338 -0.26034  C -0.55053 1.63662 -0.63899  H -0.95565 2.65855 -0.57725  H -0.33423 1.47897 -1.71328  C -1.62033 0.65843 -0.20784  C -2.62775 0.28750 -1.10781  C -1.64646 0.12455 1.08463  C -3.64271 -0.58653 -0.72249  H -2.61175 0.68864 -2.12627  C -2.65879 -0.75396 1.47154  H -0.85754 0.40036 1.78991  C -3.66172 -1.11167 0.57076  H -4.42025 -0.86549 -1.43839  H -2.66178 -1.16423 2.48477  H -4.45340 -1.80173 0.87287 | 29  M14 Energy: -421101.9358787  N 0.76276 1.29573 -0.41078  C 1.37384 0.02680 -0.23679  C 2.25707 -0.21830 0.82693  C 1.06090 -1.02276 -1.11417  C 2.82615 -1.47945 0.98434  H 2.49498 0.57573 1.53134  C 1.63525 -2.28035 -0.94367  H 0.36614 -0.86307 -1.94003  C 2.52514 -2.51726 0.10245  H 3.50976 -1.65162 1.81945  H 1.37871 -3.08187 -1.64059  H 2.97362 -3.50442 0.23468  C -0.53486 1.36678 -1.07033  H -0.80746 2.43334 -1.10153  H -0.45687 1.05842 -2.12490  C -1.61442 0.58219 -0.37456  C -1.66392 0.50387 1.02269  C -2.60870 -0.05911 -1.12128  C -2.68906 -0.19487 1.65758  H -0.88609 0.99115 1.61835  C -3.63872 -0.75364 -0.48751  H -2.57127 -0.01551 -2.21423  C -3.68140 -0.82437 0.90471  H -2.71236 -0.24943 2.74884  H -4.40705 -1.24991 -1.08563  H -4.48364 -1.37398 1.40300  C 1.37373 2.47633 -0.05821  H 0.70526 3.34114 -0.28496  O 2.47887 2.61060 0.41983 |
| M15 | 12  M15 Energy: -132576.2181416  C 1.19212 -0.34775 0.04107  H 1.97144 -0.53497 -0.72387  H 1.69325 -0.53898 1.01534  C 0.66820 1.04935 -0.01094  H 1.31301 1.93033 -0.02410  C -0.66824 1.04933 -0.01098  H -1.31308 1.93028 -0.02416  C -1.19210 -0.34779 0.04109  H -1.97142 -0.53505 -0.72385  H -1.69322 -0.53902 1.01537  N 0.00002 -1.16032 -0.18143  H 0.00004 -2.00920 0.37379 | 14  M15 Energy: -203700.2306778  C -0.20649 -1.14036 0.00162  H 0.14485 -1.70751 0.88369  H 0.14860 -1.70879 -0.87804  C -1.67918 -0.91195 -0.00119  H -2.39974 -1.73104 -0.00252  C -1.97271 0.39203 -0.00148  H -2.97317 0.82676 -0.00304  C -0.73925 1.22933 0.00119  H -0.67872 1.88958 0.88620  H -0.67577 1.89198 -0.88174  N 0.31634 0.22085 0.00143  C 1.63856 0.48366 -0.00100  H 1.84770 1.58222 -0.00160  O 2.51579 -0.36317 -0.00098 |
| M16 | 16  M16 Energy: -228214.2700267  C -0.25301 -0.67044 -0.00003  C -0.25047 0.75530 0.00000  C 0.98461 1.42890 0.00006  C 2.15865 0.68681 0.00006  C 2.13103 -0.72227 0.00001  C 0.92834 -1.41928 -0.00003  C -2.38307 0.02616 -0.00010  C -1.62058 1.16999 -0.00001  H 1.01756 2.52175 0.00010  H 3.12312 1.20096 0.00010  H 3.07217 -1.27784 0.00002  H 0.90381 -2.51163 -0.00004  H -1.99841 2.19016 0.00003  H -3.46392 -0.09687 -0.00014  N -1.56382 -1.07440 0.00004  H -1.88060 -2.03676 -0.00019 | 18  M16 Energy: -299324.6722214  C 0.00988 -0.15262 -0.00002  C -0.83927 0.98236 0.00004  C -2.22862 0.79727 0.00001  C -2.73061 -0.50034 -0.00009  C -1.87039 -1.60991 -0.00016  C -0.48494 -1.45667 -0.00013  C 1.29507 1.71758 0.00005  C 0.00525 2.14895 0.00018  H -2.90193 1.65778 0.00007  H -3.81134 -0.66138 -0.00011  H -2.29269 -2.61756 -0.00025  H 0.18418 -2.31569 -0.00020  H -0.31963 3.18710 0.00028  H 2.23279 2.26937 0.00004  N 1.32717 0.32075 0.00001  C 2.49417 -0.42258 0.00024  H 3.39122 0.23706 -0.00001  O 2.54050 -1.62827 -0.00007 |
| D1 | 18  D1 Energy: -168822.7717243  C 0.83766 1.09535 0.02669  H 1.41233 1.56916 0.84087  H 0.65790 1.89194 -0.71685  C -0.50228 0.62427 0.57532  H -1.03501 1.48901 1.03117  H -0.34373 -0.08744 1.40548  N -1.27075 -0.05031 -0.45059  H -1.44495 0.60250 -1.21446  N 1.66166 0.07941 -0.60220  H 1.13188 -0.30659 -1.38307  C 2.03154 -1.00667 0.27519  H 1.18808 -1.60942 0.67155  H 2.70329 -1.70311 -0.24775  H 2.58512 -0.61691 1.14426  C -2.52704 -0.57870 0.01978  H -3.19592 0.16785 0.49880  H -3.08541 -1.03715 -0.80868  H -2.34925 -1.36907 0.76631 | 22  D1 Energy: -311070.4914506  C -0.64525 -1.51096 -0.40551  H -1.20654 -2.43768 -0.20989  H -0.41441 -1.50549 -1.48169  C 0.64528 -1.51097 0.40551  H 1.20656 -2.43769 0.20986  H 0.41445 -1.50555 1.48169  N 1.48619 -0.37069 0.11266  N -1.48616 -0.37068 -0.11264  C -2.28069 -0.39260 1.09091  H -3.01265 -1.21423 1.06573  H -1.65653 -0.52104 1.98933  H -2.81665 0.55900 1.17315  C 2.28067 -0.39257 -1.09092  H 2.81689 0.55890 -1.17298  H 3.01239 -1.21441 -1.06593  H 1.65645 -0.52064 -1.98936  C -1.45810 0.73711 -0.90176  H -0.78671 0.58771 -1.78373  C 1.45800 0.73716 0.90170  H 0.78686 0.58763 1.78384  O -2.07901 1.76564 -0.71169  O 2.07904 1.76562 0.71173 |
| D2 | 18  D2 Energy: -168825.3990543  C 0.21773 -0.57574 0.69566  H -0.12203 -0.49052 1.75545  H 0.28876 -1.65712 0.48561  C 1.60008 0.04338 0.54714  H 2.28722 -0.47166 1.23678  H 1.58255 1.09130 0.89305  N 2.15231 0.00531 -0.79633  H 2.25483 -0.97234 -1.07226  H 1.43043 0.35560 -1.42904  N -0.75603 -0.01888 -0.22860  C -1.95477 -0.81840 -0.29164  H -2.51640 -0.85462 0.66891  H -2.64107 -0.42192 -1.05466  H -1.71135 -1.85502 -0.56731  C -1.06025 1.36686 0.03965  H -0.16235 1.99630 -0.03923  H -1.78404 1.74344 -0.69742  H -1.49727 1.53504 1.04979 | 20  D2 Energy: -239946.0886568  C -0.84886 -0.60642 0.83959  H -0.63828 -0.04954 1.76681  H -1.50144 -1.46228 1.12467  C 0.45480 -1.14303 0.28212  H 0.25946 -1.83875 -0.55388  H 0.97386 -1.73231 1.05165  N 1.31355 -0.06145 -0.13706  H 0.85887 0.71523 -0.61585  N -1.50650 0.30540 -0.08043  C 2.64288 0.01364 0.08002  H 3.01728 -0.88065 0.64192  O 3.37047 0.92336 -0.27283  C -1.98130 -0.34429 -1.28064  H -2.73434 -1.13891 -1.08326  H -2.44857 0.39471 -1.94655  H -1.15346 -0.80374 -1.83956  C -2.55424 1.05875 0.56656  H -3.39626 0.42750 0.92655  H -2.15489 1.60221 1.43490  H -2.97493 1.80000 -0.12822 |
| D3 | 12  D3 Energy: -119517.3903904  C -0.56189 0.51549 0.00008  H -0.43947 1.17538 -0.87797  H -0.43947 1.17510 0.87834  C 0.56189 -0.51549 -0.00009  H 0.43946 -1.17510 -0.87835  H 0.43948 -1.17537 0.87797  N 1.91310 0.01581 -0.00000  H 2.02575 0.63193 -0.80565  H 2.02587 0.63135 0.80607  N -1.91310 -0.01581 -0.00001  H -2.02579 -0.63182 0.80572  H -2.02582 -0.63146 -0.80601 | 16  D3 Energy: -261759.7850005  C -0.73931 1.62335 -0.18427  H -0.84889 1.61804 -1.27983  H -1.21087 2.55069 0.17245  C 0.73921 1.62337 0.18426  H 0.84879 1.61803 1.27983  H 1.21074 2.55073 -0.17244  N 1.44663 0.48221 -0.34060  H 1.67479 0.46666 -1.33243  N -1.44670 0.48217 0.34059  H -1.67499 0.46666 1.33238  C 1.69003 -0.65363 0.35657  H 1.36903 -0.55250 1.42535  O 2.20520 -1.65948 -0.08961  C -1.68993 -0.65373 -0.35655  H -1.36885 -0.55262 -1.42531  O -2.20511 -1.65958 0.08961 |
| D4 | 15  D4 Energy: -144170.8002626  C -0.05838 0.73589 0.23179  H -0.38904 1.76438 0.00237  H -0.10803 0.65132 1.34473  C 1.37854 0.55156 -0.20745  H 1.99533 1.36496 0.22479  H 1.43051 0.67223 -1.30210  N 1.83743 -0.78969 0.12264  H 2.75870 -0.94885 -0.28115  H 1.97807 -0.85666 1.13116  N -0.92263 -0.21135 -0.43555  H -0.48507 -1.12864 -0.34780  C -2.25996 -0.24753 0.09544  H -2.77229 0.71159 -0.08496  H -2.85256 -1.02651 -0.40521  H -2.32040 -0.43610 1.18987 | 19  D4 Energy: -286415.1377957  C -0.58050 -1.45154 -0.62934  H -1.18511 -2.35547 -0.45454  H -0.51459 -1.31880 -1.72023  C 0.81694 -1.64647 -0.05326  H 1.25190 -2.57538 -0.44885  H 0.76416 -1.77166 1.03923  N 1.69507 -0.53846 -0.33625  H 2.07937 -0.45306 -1.27479  N -1.26179 -0.30346 -0.07271  C -1.82663 -0.41303 1.24983  H -2.29110 0.54392 1.51081  H -2.59070 -1.20426 1.28756  H -1.05919 -0.64467 2.00523  C -1.30460 0.87889 -0.74474  H -0.81894 0.78998 -1.74785  C 1.89569 0.50766 0.50066  H 1.39360 0.34160 1.48880  O 2.54908 1.50033 0.24742  O -1.80754 1.91070 -0.34262 |
| D5 | 21  D5 Energy: -193478.4683614  C -0.47059 -0.89487 0.52185  H -0.95111 -1.09906 1.50617  H -0.50425 -1.83981 -0.04493  C 0.97628 -0.50769 0.74201  H 1.48613 -1.33347 1.26893  H 1.03651 0.35864 1.44138  N 1.63321 -0.25317 -0.51987  H 1.01496 0.33690 -1.07649  N -1.20793 0.09664 -0.24515  C -2.49225 -0.40472 -0.66895  H -2.36965 -1.33505 -1.24237  H -3.18351 -0.62452 0.17521  H -2.99406 0.32614 -1.31995  C -1.32829 1.36597 0.43265  H -0.34132 1.81538 0.61586  H -1.89280 2.07233 -0.19257  H -1.85205 1.29714 1.41235  C 2.92596 0.36465 -0.38411  H 2.92785 1.30935 0.20308  H 3.62853 -0.31853 0.12017  H 3.35117 0.59025 -1.37234 | 23  D5 Energy: -264601.4578081  C -1.13019 -0.75520 0.75893  H -1.83513 -0.75782 1.62274  H -1.17751 -1.77366 0.33862  C 0.26713 -0.49966 1.29965  H 0.53200 -1.32938 1.97854  H 0.29941 0.41613 1.90921  N 1.26607 -0.36660 0.26097  N -1.52898 0.16757 -0.28280  C 2.01817 0.75626 0.14480  H 1.77967 1.48817 0.95637  O 2.86043 0.96903 -0.70858  C -1.61424 1.53520 0.16887  H -0.64513 1.89305 0.54541  H -1.89478 2.19031 -0.66808  H -2.36417 1.68918 0.97718  C -2.74286 -0.25739 -0.93536  H -2.62477 -1.27466 -1.33744  H -3.63103 -0.26651 -0.26454  H -2.97720 0.40939 -1.77794  C 1.39828 -1.43154 -0.70093  H 1.49076 -2.40406 -0.19458  H 0.53019 -1.47253 -1.37848  H 2.29685 -1.25267 -1.30130 |
| D6 | 19  D6 Energy: -216033.7031529  N -0.02355 0.31637 -0.39203  H 0.59755 0.89511 -0.95617  C 0.76114 -0.22526 0.70062  H 0.92998 0.52725 1.50495  H 0.22001 -1.05926 1.17926  C 2.09893 -0.71963 0.19175  H 1.92207 -1.49986 -0.56679  H 2.63818 -1.21390 1.02394  C -1.20433 1.05816 0.00571  H -1.54244 1.64340 -0.86411  H -1.01776 1.77898 0.82998  C -2.29843 0.08391 0.40631  H -3.25700 0.61558 0.52315  H -2.06691 -0.34963 1.40347  O -2.44049 -0.91439 -0.56668  H -1.53946 -0.99719 -0.93019  N 2.83079 0.36764 -0.43944  H 3.18249 0.99972 0.28022  H 3.66274 0.00375 -0.90025 | 23  D6 Energy: -358277.4783647  N 1.32553 0.36413 0.33082  C 0.93862 -0.78814 1.13553  H 1.83916 -1.27178 1.54648  H 0.34820 -0.41722 1.98567  C 0.13281 -1.81618 0.34591  H -0.28453 -2.56857 1.03021  H 0.79104 -2.34263 -0.35849  C 0.80735 1.68555 0.64633  H 1.32868 2.40197 -0.00616  H 1.07080 1.95346 1.68367  C -0.68658 1.83860 0.46995  H -0.96544 2.84794 0.83047  H -1.21616 1.13119 1.13661  O -1.05388 1.65263 -0.87432  H -1.92862 1.22016 -0.87755  N -0.94658 -1.19393 -0.38423  H -0.67041 -0.47361 -1.05505  C 2.25622 0.19271 -0.64283  H 2.47957 1.14768 -1.17804  C -2.21306 -1.09283 0.05867  H -2.45097 -1.82484 0.86703  O 2.79569 -0.86533 -0.91394  O -3.04258 -0.30147 -0.37577 |
| D7 | 26  D7 Energy: -312548.3420451  C 0.66909 -1.61789 -0.17140  H 0.55730 -1.94552 -1.23012  H 1.27382 -2.39897 0.31836  C -0.69751 -1.56144 0.47197  H -1.13932 -2.57930 0.48593  H -0.59163 -1.25632 1.52697  N -1.54582 -0.58801 -0.19272  H -1.67144 -0.87005 -1.16664  N 1.35073 -0.34585 -0.05434  H 0.71670 0.38381 -0.38376  C 2.61009 -0.26038 -0.76838  H 2.50497 -0.28846 -1.87318  H 3.21669 -1.13966 -0.49038  C 3.32844 0.99822 -0.33127  H 4.33749 1.04032 -0.77099  H 2.78221 1.88411 -0.72363  C -2.85159 -0.40758 0.41749  H -3.50467 -1.30190 0.34738  H -2.69578 -0.21741 1.49301  C -3.52653 0.79247 -0.20916  H -4.48145 1.00319 0.29733  H -3.78207 0.55720 -1.26563  O -2.71166 1.93066 -0.11787  H -1.81340 1.58287 -0.24451  O 3.44360 1.04139 1.06560  H 2.60875 0.64627 1.37192 | 30  D7 Energy: -454794.4298540  C 0.49351 0.43243 -1.13908  H -0.13630 1.33070 -1.14498  H 1.24264 0.54860 -1.93643  C -0.35341 -0.79939 -1.42186  H -0.76348 -0.72807 -2.43907  H 0.28459 -1.69736 -1.41023  N -1.44671 -0.99644 -0.48972  N 1.17734 0.35125 0.14279  C 2.40205 -0.42430 0.22583  H 2.29959 -1.33812 -0.38062  H 2.53564 -0.75683 1.26777  C 3.62781 0.35355 -0.22849  H 3.47979 0.71806 -1.25716  H 3.74194 1.25512 0.40529  C -2.78225 -0.48356 -0.77683  H -2.85778 -0.33969 -1.86473  H -3.50982 -1.25966 -0.49095  C -3.14388 0.80571 -0.06302  H -3.07510 0.64221 1.02799  H -4.20726 1.01185 -0.27654  O -2.38775 1.91260 -0.46974  H -1.61406 1.95746 0.12448  O 4.78065 -0.44307 -0.24376  H 4.98969 -0.67498 0.67038  C 0.71536 1.00697 1.22999  H 1.35222 0.82538 2.12604  C -1.25574 -1.71669 0.64568  H -0.21140 -2.11325 0.71769  O -0.27287 1.72704 1.28062  O -2.10577 -1.93576 1.48990 |

# **References**

1. Pracht, P., F. Bohle, and S. Grimme, *Automated exploration of the low-energy chemical space with fast quantum chemical methods.* Physical Chemistry Chemical Physics, 2020. **22**(14): p. 7169-7192.

2.  *Gaussian 16, Revision C.01,*

*M. J. Frisch, G. W. Trucks, H. B. Schlegel, G. E. Scuseria,*

*M. A. Robb, J. R. Cheeseman, G. Scalmani, V. Barone,*

*G. A. Petersson, H. Nakatsuji, X. Li, M. Caricato, A. V. Marenich,*

*J. Bloino, B. G. Janesko, R. Gomperts, B. Mennucci, H. P. Hratchian,*

*J. V. Ortiz, A. F. Izmaylov, J. L. Sonnenberg, D. Williams-Young,*

*F. Ding, F. Lipparini, F. Egidi, J. Goings, B. Peng, A. Petrone,*

*T. Henderson, D. Ranasinghe, V. G. Zakrzewski, J. Gao, N. Rega,*

*G. Zheng, W. Liang, M. Hada, M. Ehara, K. Toyota, R. Fukuda,*

*J. Hasegawa, M. Ishida, T. Nakajima, Y. Honda, O. Kitao, H. Nakai,*

*T. Vreven, K. Throssell, J. A. Montgomery, Jr., J. E. Peralta,*

*F. Ogliaro, M. J. Bearpark, J. J. Heyd, E. N. Brothers, K. N. Kudin,*

*V. N. Staroverov, T. A. Keith, R. Kobayashi, J. Normand,*

*K. Raghavachari, A. P. Rendell, J. C. Burant, S. S. Iyengar,*

*J. Tomasi, M. Cossi, J. M. Millam, M. Klene, C. Adamo, R. Cammi,*

*J. W. Ochterski, R. L. Martin, K. Morokuma, O. Farkas,*

*J. B. Foresman, and D. J. Fox, Gaussian, Inc., Wallingford CT, 2019.*

3. Neese, F., et al., *The ORCA quantum chemistry program package.* The Journal of Chemical Physics, 2020. **152**(22).

4. Safy, M.E., et al., *Kinetic Modeling of the Ru‐MACHO‐Catalyzed CO2 Hydrogenation to Methanol.* ChemCatChem: p. e00883.

5. Hoops, S., et al., *COPASI--a COmplex PAthway SImulator.* Bioinformatics, 2006. **22**(24): p. 3067-74.

6. Jayarathne, U., N. Hazari, and W.H. Bernskoetter, *Selective Iron-Catalyzed N-Formylation of Amines using Dihydrogen and Carbon Dioxide.* ACS Catalysis, 2018. **8**(2): p. 1338-1345.

7. Safy, M.E.A., et al., *Kinetic Modeling of the Ru-MACHO-Catalyzed CO2 Hydrogenation to Methanol.* ChemCatChem. **n/a**(n/a): p. e00883.

8. Kar, S., et al., *Mechanistic Insights into Ruthenium-Pincer-Catalyzed Amine-Assisted Homogeneous Hydrogenation of CO(2) to Methanol.* J Am Chem Soc, 2019. **141**(7): p. 3160-3170.
